# Early Transcriptome Analyses of Z-3-Hexenol-Treated *Zea mays* Revealed Distinct Transcriptional Networks and Anti-Herbivore Defense Potential of Green Leaf Volatiles

**DOI:** 10.1371/journal.pone.0077465

**Published:** 2013-10-14

**Authors:** Jurgen Engelberth, Claudia Fabiola Contreras, Chinmay Dalvi, Ting Li, Marie Engelberth

**Affiliations:** Department of Biology, University of Texas at San Antonio, San Antonio, Texas, United States of America; Centro de Investigación y de Estudios Avanzados, Mexico

## Abstract

Green leaf volatiles (GLV), which are rapidly emitted by plants in response to insect herbivore damage, are now established as volatile defense signals. Receiving plants utilize these molecules to prime their defenses and respond faster and stronger when actually attacked. To further characterize the biological activity of these compounds we performed a microarray analysis of global gene expression. The focus of this project was to identify early transcriptional events elicited by *Z*-3-hexenol (*Z*-3-HOL) as our model GLV in maize (*Zea mays*) seedlings. The microarray results confirmed previous studies on *Z*-3-HOL -induced gene expression but also provided novel information about the complexity of *Z*-3-HOL -induced transcriptional networks. Besides identifying a distinct set of genes involved in direct and indirect defenses we also found significant expression of genes involved in transcriptional regulation, Ca^2+^-and lipid-related signaling, and cell wall reinforcement. By comparing these results with those obtained by treatment of maize seedlings with insect elicitors we found a high degree of correlation between the two expression profiles at this early time point, in particular for those genes related to defense. We further analyzed defense gene expression induced by other volatile defense signals and found *Z*-3-HOL to be significantly more active than methyl jasmonate, methyl salicylate, and ethylene. The data presented herein provides important information on early genetic networks that are activated by *Z*-3-HOL and demonstrates the effectiveness of this compound in the regulation of typical plant defenses against insect herbivores in maize.

## Introduction

The response of plants to insect herbivore damage is characterized by a massive reprogramming of metabolic pathway aiming towards the reduction of damage. This reprogramming is initiated immediately after an insect starts feeding on a plant. Besides causing physically damage, which is often already sufficient to initiate effective defense signaling, insect herbivores often also apply elicitors abundant in their saliva to the damage site resulting in the production of jasmonic acid (JA) [1-5]. Among the elicitors known to date fatty acid-amino acid conjugates like volicitin and N-linolenoyl-glutamine are probably the best characterized and exhibit the most widespread activity within the plant kingdom [5,6]. A common element among all these insect elicitors (IE) is the fact that they induce JA in plants above the mere wounding level and also often signal damage to other parts of the plant [1-4,6]. 

JA is produced through the octadecanoid signaling pathway beginning by the incorporation of molecular oxygen into α-linolenic acid by a lipoxygenase in the chloroplast. After being converted by allene oxide synthase and allene oxide cyclase into 9*S*, 13*S-*12-oxo phytodienoic acid (or *cis*-OPDA), this intermediate is then transported into the peroxisome, where the pentacyclic system is reduced and after three cycles of β-oxidation JA is released into the cytosol. There, JA is conjugated to an amino acid, for example isoleucine [7,8]. JA-Ile then binds to its receptor COI1 [9], which is an essential part of a SCF-protein complex. The targets for this complex are JAZ proteins, which act as suppressor of JA-activated transcription factors like MYC2 in *Arabidopsis* [9,10]. Once MYC2 is released form it JAZ suppressor it initiates the transcription of typical JA-inducible genes [10]. 

The major activity for JA-Ile signaling lies in the massive reprogramming of metabolic pathways [11], in particular those responsible for resistance against the attacking insect herbivore. Among those metabolic changes are the production of toxic secondary metabolites, the production of proteins that reduce the nutritional value of the consumed plant material, and the production of so-called herbivore-induced plant volatiles (HIPV). HIPV comprise a complex mixture of metabolites from various pathways like terpenes, shikimic-derived products, as well as fatty acid-derived products, that are released rapidly first from the damaged parts of the plants, but later also from undamaged, systemic parts of the same plant [12,13]. HIPV play important roles as mediators of tritrophic interactions, for example by attracting natural enemies of the attacking insect herbivore and/or by serving as a repellent [14-19]. Another function for these volatiles became apparent in recent years, when it was discovered that undamaged neighboring plants can “smell” some of these compounds that are emitted by damaged plants [20-22]. This communication between plants through the release of volatiles was first described by 20,21. Both found independently that plants exposed to volatiles from damaged neighboring plants became less attractive to insect herbivores. More than 15 years later it was found that plants exposed to volatiles from herbivore-infested plants accumulate transcripts of defense genes that were previously shown to be important in the insect herbivore defense [22]. 

To date several individual components of the often-complex blends of HIPV have been described to induce genes related to insect herbivore defense responses in various plant species. Methyl jasmonate (MeJA), several terpenes like 3*E*-4,8-dimethyl-l,3,7-nonatriene (DMNT) and ocimene, *cis*-jasmone, as well as green leaf volatiles (GLV) have been shown to significantly affect plants exposed to these compounds by fortifying their defenses [22-29]. However, while the composition of HIPV varies enormously, GLV have emerged as volatile signaling compounds that are common to all plant species [30,31]. 

GLV are released immediately in significant amounts by plants when under insect herbivore attack [12-14]. In contrast to other HIPV like terpenes, which are synthesized *de novo*, GLV are produced from existing precursors, which makes them ideal rapid and universal candidates for volatile-mediated inter- and intra-plant signaling. The biosynthetic pathway for GLV starts with linolenic acid, which is oxygenated by a lipoxygenases (LOX). The resulting 13-hydroperoxy-linolenic acid is then catalyzed by the enzyme hydroperoxide lyase (HPL). Major products of this pathway are *Z*-3-hexenal, *Z*-3-hexenol (*Z*-3-HOL), and *Z*-3-hexenyl acetate and their respective *E*-2-enantiomers [30,31]. Although the HPL pathway was already characterized 100 years ago [30], it has only recently gained significance, when it was shown that the volatile products of this pathway serve as potent signals in inter- and intra-plant signaling. For example, Bate and Rothstein [32] demonstrated that individual members of this family of volatiles, when applied as pure chemicals, induced defense-related genes in *Arabidopsis*. However, these responses were always incomplete or less prominent when compared to actual herbivory and raised questions about the effectiveness of this kind of volatile signaling. In a study by [33] it was shown that GLV may have a function apart from just providing direct protection. Maize seedlings that were previously exposed to GLV from neighboring plants produced significantly more JA and volatile sesquiterpenes upon subsequent treatment with IE when compared to appropriate controls. This was the first report on priming against insect herbivory signaled by GLV and it was demonstrated that this effect is specifically linked to anti-herbivore defenses since responses to mechanical wounding alone were not affected.

Since its initial discovery, the priming effect of GLV has been confirmed in a more natural environment [23]. By using a microarray enriched in tobacco genes related to insect herbivory, this study showed increased transcriptional responses in the plants growing adjacent to clipped sagebrush. Although no immediate increases in direct defenses like nicotine or proteinase inhibitors were found upon volatile exposure, subsequent feeding by *Manduca sexta* caterpillars, these primed plants showed an accelerated production of trypsin proteinase inhibitor. Consequently, the primed state of these tobacco plants also resulted in lower herbivore damage and higher mortality rate of young *Manduca* caterpillars. Among the volatiles responsible for this priming effect were *E*-2-hexenal, methacrolein and methyl jasmonate. Likewise, the priming effect of HIPV on direct and indirect defenses against herbivory in maize was further demonstrated by [24] on a molecular, chemical, and behavioral level. By using a differential hybridization screen they identified 10 defense-related genes, which were inducible by caterpillar feeding, mechanical wounding, application of ie, and JA. Exposure to volatiles from herbivore-infested plants did not activate these genes directly, but primed a subset of them for stronger and/or earlier induction upon subsequent defense elicitation, resulting in reduced caterpillar damage and increased attraction to the natural enemies of the caterpillar. 

Although GLV received most attention for their potential role in inter-plant signaling, other studies revealed that HIPV also serve as signals in intra-plant communication. In a study by [25] on the role of volatiles as inducers of resistance between different branches of sagebrush (*Artemisia tridentata*) it was found that airflow was essential for the induction of induced resistance in systemic tissues. A similar effect was observed for lima beans (*Phaseolus lunatus*) and the induction of extrafloral nectar. Besides providing a signal for neighboring plants, infested plants may very well send a volatile signal to other parts of themselves [26,27], thereby reducing further damage. 

The importance of the GLV pathway in the plant defense response was further confirmed by studies using either LOX- or HPL-depleted plants, which showed increased susceptibility to insect herbivory [34-37]. These studies also provided first evidence that the pathway for GLV production is directly involved in the regulation of effective defense responses against different kinds of insect herbivory including chewing and phloem-feeding insects. 

The priming of plant defense responses, which results in an accelerated and/or enhanced reaction when actually attacked, is well established [38]. Although precisely how priming agents regulate subsequent responses is unknown, they appear to work through one or more of the commonly involved major signaling pathways. For GLV-activated priming it seems clear that a functioning JA signaling pathway is required [37]. But while in maize and other monocots JA accumulates during the initial exposure to these compounds no such effect has been reported for dicot plants albeit the fact that they also recognize these volatile signals and in most cases this recognition primes JA-regulated defense responses or even the responses to JA itself [39]. Considering the conserved nature of the GLV signal emission among most, if not all plant species, it can be hypothesized that common signaling mechanisms exist for the perception of GLVs, but these have yet to be discovered. 

The study presented herein was therefore initiated to gain further insight into the early gene regulatory networks that are activated by GLV specifically within 1 hour. We selected *Z*-3-HOL as our “model” compound since it is emitted by most plants after damage, but does not have the aldehyde function of its precursor, Z-3-hexenal, which may cause unspecific responses due to its reactivity. Also, in a study by [40] is was shown that plants reduce the aldehyde to the alcohol in undamaged tissue making it more likely that *Z*-3-HOL is the essential GLV. We identified distinct levels of genetic activation coupled to significant induction of typical anti-herbivore defenses. We also found that among the common volatile defense signals including MeJA, methyl salicylate (MeSA), and ethylene (E) only *Z*-3-HOL showed such an activity in maize. Together, the data presented herein provides evidence for the potential and importance of GLV to activate defensive measures in response to insect herbivory. 

## Results

The global gene expression analysis of 2-week old *Zea mays* cv. B73 leaves after volatile exposure to *Z*-3-HOL was performed by using the 46K array provided by the University of Arizona Maize Microarray Project, which covers nearly the whole maize genome. Leaves were collected 20 and 60 min after exposure since the focus of this study was on early transcriptional changes induced by GLV based on our previous finding that showed maximum JA accumulation after *Z*-3-HOL exposure between 10-30 min after exposure [41]. For better identification we added the operon oligo identifiers in brackets behind each named gene.

Based on the selection criteria described in *Material and Methods* we identified 20 genes with a ≥ 2fold increase or decrease in transcript levels 20 min after exposure to *Z*-3-HOL (Table S1). Among those 20 genes 11 were categorized as being involved in signaling, mostly as transcription factors (8), while 2 genes were related to phosphorylation and Ca^2+^-signaling (EF-hand Ca^2+^-binding protein CCD1 (CBP CCD1, MZ00016998)) and one gene, metallothionein (MZ00042093), may be involved in the binding of xenobiotics. The 8 remaining genes were annotated as “unknown”. While 19 genes showed a significant increase ranging from 2-fold to 4-fold maximum (e.g. ethylene responsive elements like-protein (EREBP, MZ00031018), only one gene of unknown function showed a significant decrease. At this time point no genes directly or indirectly involved in defense were found to accumulate. 

Exposure of maize leaves for *Z*-3-HOL for 60 min revealed 152 significantly up- or down-regulated genes (Table S2). As it was found for the 20 min exposure time more than 40% of those genes fall into the “unknown” category. Besides genes involved in general metabolism we identified among those genes with a putative function several categories that may play important roles in GLV-induced defense responses. For example, about 17% percent of the induced genes were putatively involved in signaling, mostly transcription factors. Among those we identified another EREBP (MZ00018574) as well as MYC7 MZ00019886), a putative ortholog of MYC2 of *Arabidopsis*. Other genes involved in lipid signaling included allene oxide synthase (AOS, MZ00044190)), lipoxygenase 5 (LOX5, MZ00015701)), lipase 1 (MZ00026739), and hydroperoxide lyase (HPL, MZ00017601). We also identified 2 putative JAZ genes (MZ00014350, MZ00044135), which may act in this context as suppressors of JA-MYC7-mediated gene expression. Additionally, genes involved in Ca^2+^ signaling including calmodulin (MZ00019632) and several calcium-dependent protein kinases (MZ00050523, MZ00028280) as well as a MAPK6 (MZ00018837) were also found to be significantly upregulated at this time point. 

Besides genes putatively involved in defense signaling we also identified an array of genes with direct or indirect defensive properties at this time point. For example, we found several proteinase inhibitors like Bowman-Burk proteinase inhibitor (14-fold increase, MZ00037085), phytocystatin (MZ00029560), subtilisin/chymotrypsin inhibitor (MZ00041005), ribosome inactivating protein (RIP, MZ00042678), and maize proteinase inhibitor (MIP, MZ00033310) among others to be significantly increased. Likewise, genes involved in the production of volatiles as an indirect defensive measure like sesquiterpene cyclase 1 (13-fold increase, MZ00031736), 1-deoxyxylulose-5-phosphate synthase (MZ00032136), terpene synthase (MZ00030501), linalool synthase (MZ00019729), and a putative salicylic acid carboxyl methyltransferase (MZ00032043) were found to be significantly upregulated at this time. Interestingly, we also identified several genes involved in cell wall strengthening including proline-rich proteins (MZ00036743, MZ00041634, MZ00042242), glycine-rich proteins (MZ00043232) as well as a gene putatively involved in lignin biosynthesis, cinnamoyl-CoA reductase (MZ00023228). On the other side, transcript levels for a β-expansin (MZ00018192), which is essential for cell wall loosening during cell expansion, were significantly down. A glutathion-S-transferase (MZ00025768) was found to be among the most highly upregulated genes at this time point and may, like metallothionein, be involved in the binding of xenobiotics including *Z*-3-HOL itself [42].

**Figure 1:**
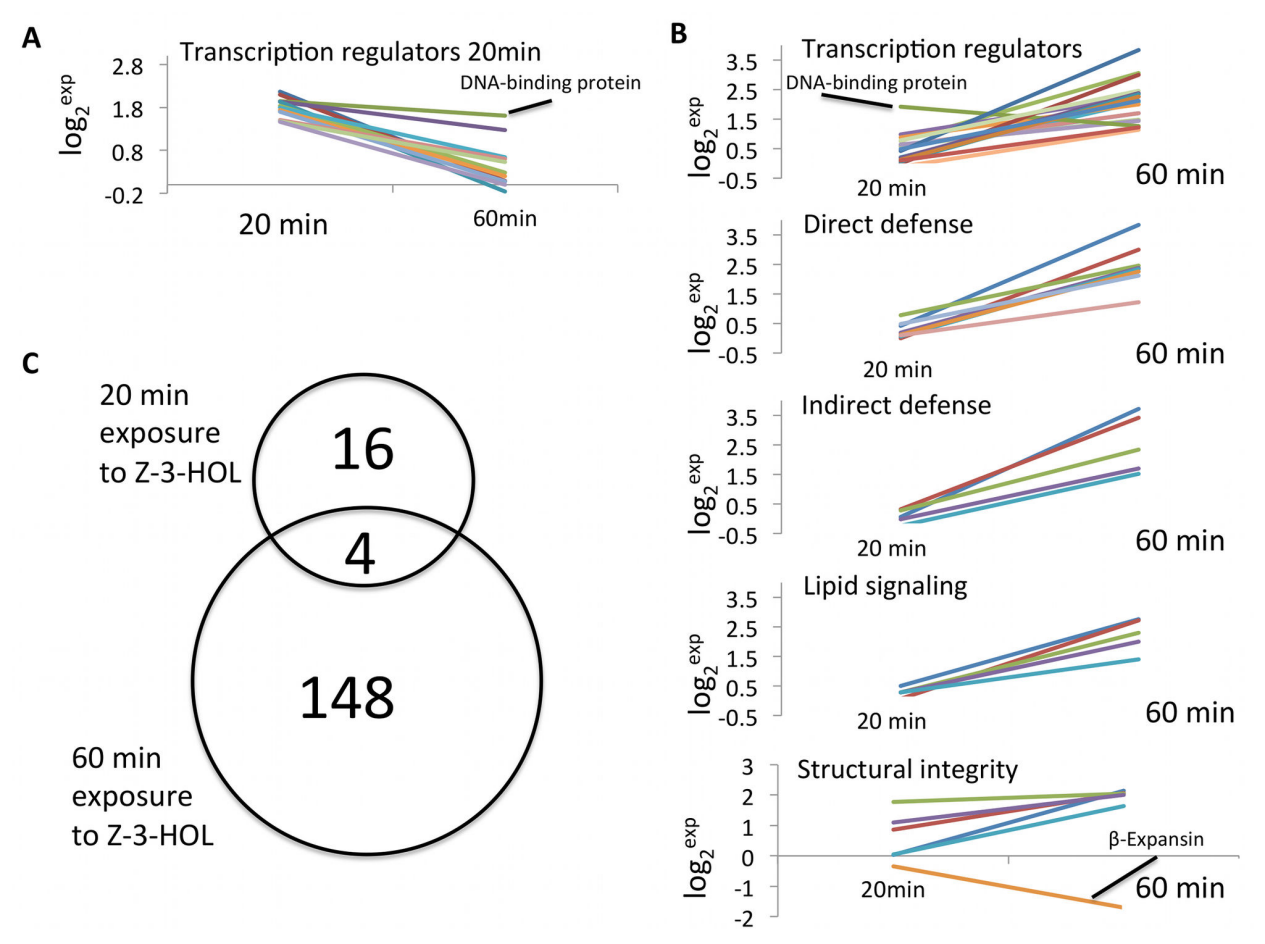
Microarray expression analysis after 20 and 60 min of exposure to Z-3-hexenol. A, time course of transcription factors (TF) with maximum expression after 20 min of exposure. B, time course of genes putatively involved in transcription, direct defense, indirect defense, lipid signaling, and maintenance of structural integrity with maximum expression after 60 min of exposure. Among those groups only β-expansin showed a significant reduction 60 min after exposure (structural integrity panel). Numbers displayed show log_2_-based increases. For A and B, averages of 3 independent microarrays are shown. All genes showed a significant change in expression of ≥2-fold (p≤0.05). C, overlap of gene expression between 20 and 60 min of exposure.

As described above, we identified 20 genes that are significantly (≥2-fold) upregulated 20 min after exposure to *Z*-3-HOL, and a total of 152 genes that showed significant increases 60 min after exposure to this volatile compound. We could only identify 4 genes that overlapped in their expression level over this time course including a helix-loop-helix DNA binding protein (MZ00005265) and 3 genes of unknown function (Figure 1C). By looking at the time course of genes that are differentially expressed at the selected time points, in particular transcription regulators, it became obvious that during exposure to *Z*-3-HOL very distinct sets of these regulators are expressed in a temporal manner. Only the above-mentioned helix-loop-helix DNA binding protein was found to be significantly up over the time course covered by this study (Figure 1A, B), strongly suggesting the existence of several tiers of transcriptional regulation of *Z*-3-HOL-induced responses. Genes involved in direct or indirect defenses, lipid signaling, as well as those with a function in cell wall reinforcement were only significantly up at 60 min after exposure (Figure 1B). 

To confirm the results obtained by the microarray analysis we performed a first PCR-based study of 6 genes selected from the microarray results in response to the same treatment in the same maize B73 inbred line. We selected allene oxide synthase (AOS, MZ00044190), ribosome-inactivating protein (RIP, MZ00042678), lipase 2 (Lipase 2, MZ00036047), and the transcription factors WRKY12 (MZ00042508), EREBP 4 (MZ00031018), and MYC7 (MZ00019886) (seeTable 1). We performed a quantitative PCR analysis of these genes with GapC as the internal standard (see *Material and Methods*). The selected genes were not all chosen from those significantly upregulated in the microarray, but were also selected from those showing a non-significant increase in transcript level. This was done to not only confirm the array results, but also to test for the overall quality of the array. 

**Table 1:** Genes selected for PCR-based expression analyses with primer sequences.

**Name/ Identifier(ID)**	**Putative annotation**	**Primer 5’ - 3’**	**Sequence**	**Microarray fold increase**
TC261915/ MZ00016998	EF-hand calcium binding protein CCD1	forward/ reverse	CACCAGCTGATGATGACGAC/ ATGGGAATTGGGAAGGAAAG	3.56 at 20min
TC277131/ MZ00031018	EREBP-like protein 4	forward/ reverse	GTGGTGGTGCGATCCTCGCC/ GGATCTCGGCGGCCCACTTG	2.46 at 20min
TC260428/ MZ00042508	Transcription factor WRKY12	forward/ reverse	GCCAAGCGCTGGAAGCAGGA/ GGCCATCAAGGCCCCGGAAC	1.86 at 20min
TC202729/ MZ00042678	Ribosome inactivating protein (RIP)	forward/ reverse	CCCGTGGAGGACACGGCCTA/ TGTCGCCGTCCTTGCCGAAC	4.78 at 60min
TC248865/ MZ00019886	Transcription factor MYC7	forward/ reverse	GTCTGCTTCCCCGTCGGCAC/ GCGTCGGCGAGCCATAGCAT	2.80 at 60min
AZM4_22891/ MZ00036047	Putative lipase (Lipase 2)	forward/ reverse	TATACAAGACGCCGGGAGAC/ GATCTGTGGTGCCGTCTGT	2.30 at 60min
TC271827/ MZ00044190	Allene oxide synthase (AOS)	forward/ reverse	GACCGCCTCGACTTCTACTAC/ GAAGAGCAGCTGCTTCACCTT	4.02 at 60min
TC260211/ MZ00024620	Allene oxide cyclase (AOC)	forward/ reverse	AAGGTGCAGGAGCTGTACG/ CAGGTACGACTCCTCGTAGGT	1.56 at 60min
TC270178/ MZ00019632	Calmodulin-like	forward/ reverse	AATAATCCCGCCTCGTATCC/ GTCGAAGAGGTCGAACACG	2.84 at 60min

Displayed are also the fold-increases for the respective genes in the microarray study after high Z-3-hexenol exposure. Selection of genes for this study was based on the temporal expression pattern and their putative involvement in cellular signaling, transcriptional regulation, and defense.

**Figure 2:**
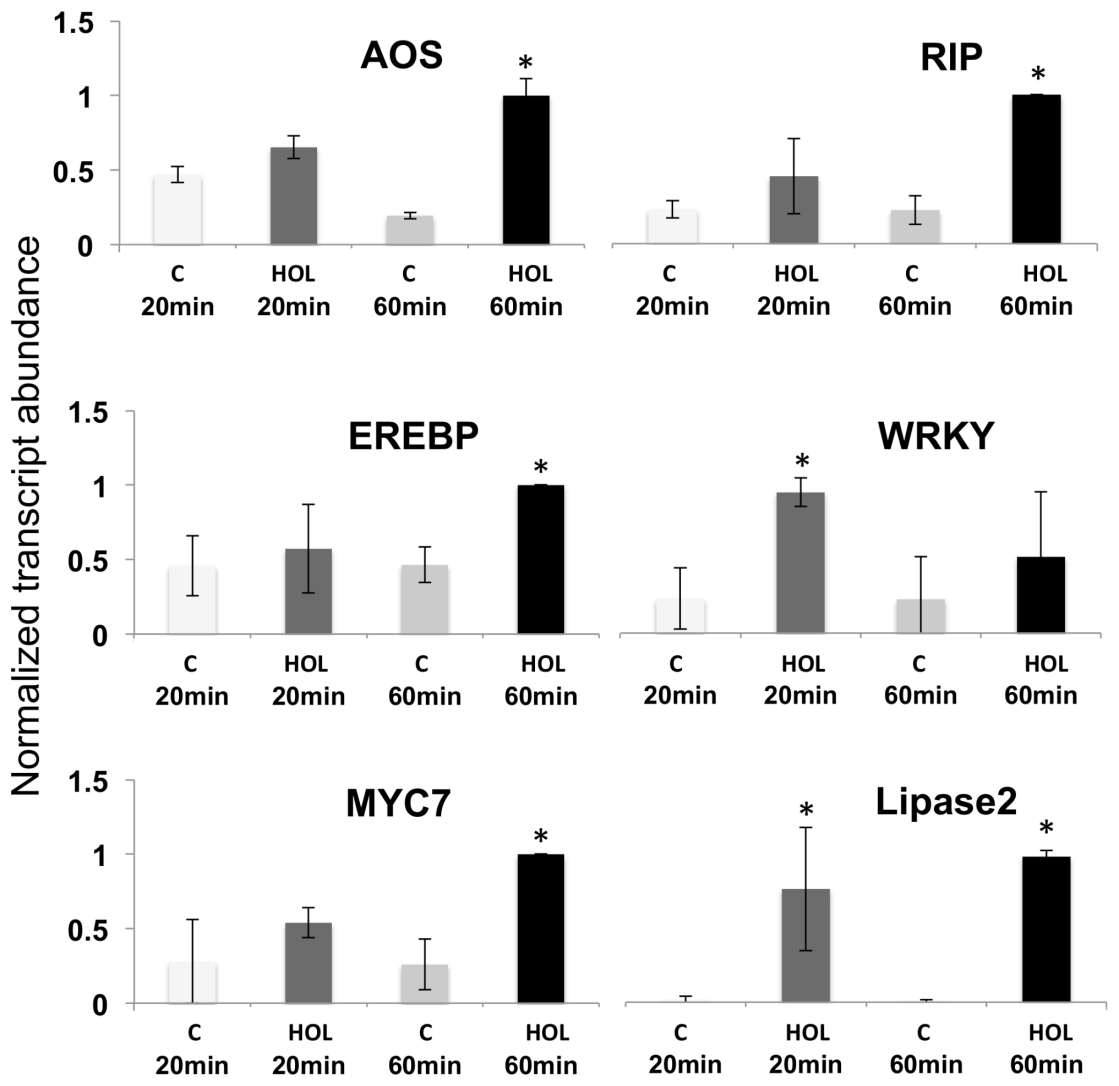
Confirmation of microarray results with selected genes by quantitative PCR. Gene expression is displayed as relative abundance of transcripts in maize leaves (Inbread line B73) after exposure to Z-3-hexenol (app. 1.4 µM in the gas phase) for 20 and 60 min. Data is expressed as the gene/GapC ratio. Data were normalized. All experiments have been performed with at least three biological replicates. A Student’s *t*-test was used for proof of significance (*, p≤0.05) when compared to the respective control. The lines on each bar represent standard deviation. Abbreviations: AOS, allene oxide synthase; RIP, ribosome inactivating protein; EREBP, ethylene response element binding protein.

Among the 6 genes tested by PCR at high *Z*-3-HOL (as used in the microarray study) we found 5 of them following the same temporal pattern established by the microarray. AOS, RIP, MYC7, and lipase 2 showed maximum transcript accumulation 60 min after exposure, and WRKY12 a maximum at 20 min (Figure 2). Only EREBP, which on the microarray showed a 2.46-fold increase 20 min after treatment, had its maximum transcript accumulation in the PCR confirmation at 60 min (2.2-fold increase) (Figure 2, Table 1). 

**Figure 3:**
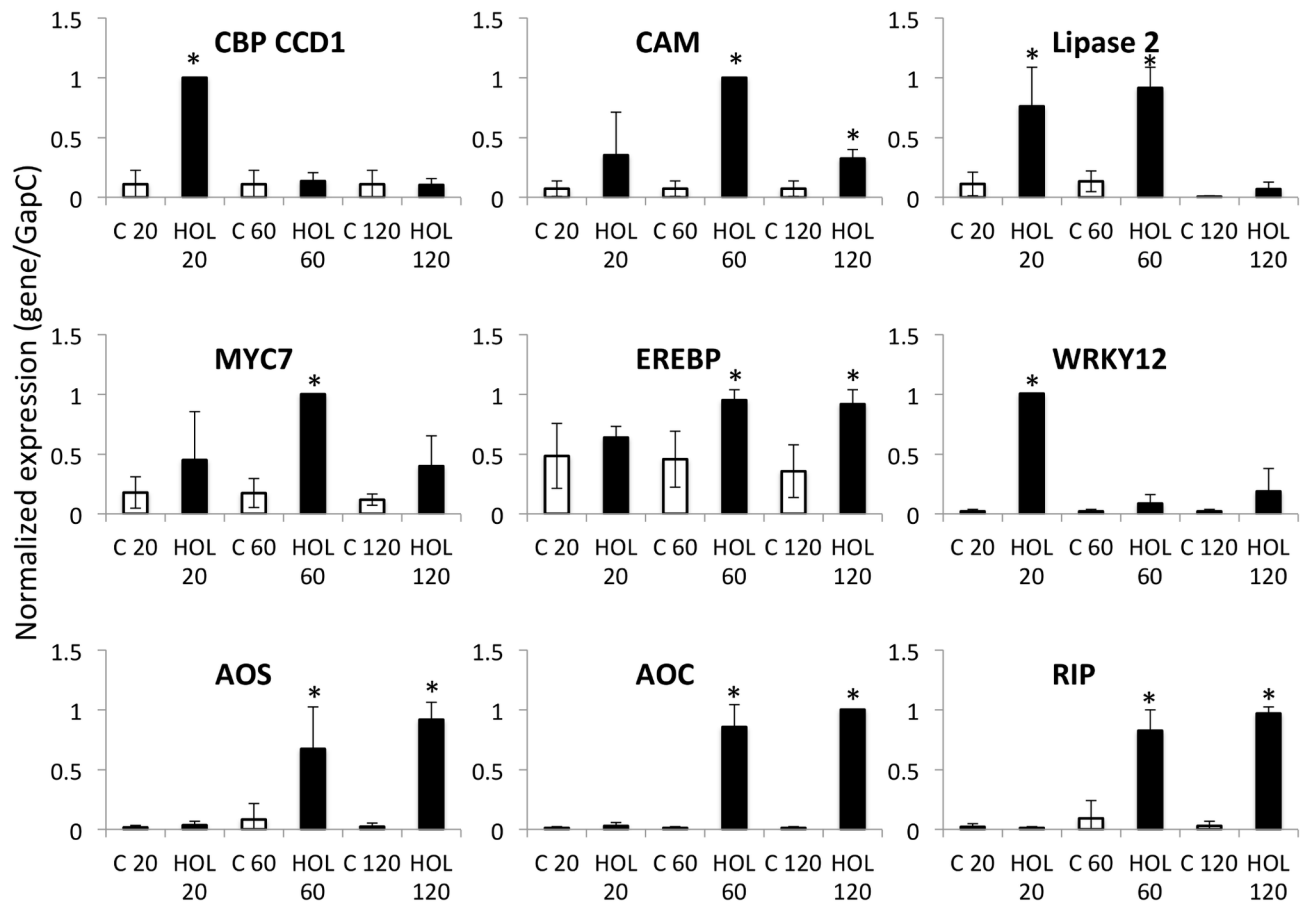
Analysis of gene expression of selected genes (see Table 1) by PCR after exposure to low concentrations of *Z*-3-hexenol. Gene expression is displayed as relative abundance of transcripts in maize leaves (cv “Kandy King”) after exposure to Z-3-hexenol (app. 14 nM in the gas phase) for 20, 60, and 120 min. Data is expressed as the gene/GapC ratio. All data were normalized. All experiments have been performed with at least three biological replicates. A Student’s *t*-test was used for proof of significance (*, p≤0.05) when compared to the respective control. The lines on each bar represent standard deviation. Abbreviations: C, control; HOL, Z-3-hexenol; AOS, allene oxide synthase; AOC, allene oxide cyclase; CBP CCD1, EF-hand Ca^2+^-binding protein CCD1; CAM, calmodulin; EREBP, ethylene response element binding protein; RIP, ribosome inactivating protein.

While the concentration of *Z*-3-HOL used in the above-described microarray study has to be considered too high to occur in nature as a means of interplant communication caused by insect herbivory it may mimic the situation at and around the site of actual damage. We therefore performed an additional study with a100-fold reduced concentration of *Z*-3-HOL (approximately 14 nM maximum concentration in the gas phase) that matches more accurately the amounts of GLV which are released by maize seedling in response to mechanical damage and insect herbivory and may be received by neighboring plants [33]. Additionally, we used an “open” system, in which the glass cylinders containing the maize seedlings for exposure to *Z*-3-HOL were placed on spacers to allow for a better gas exchange and therefore reduced some stresses caused by closed systems. Also, we selected another maize cultivar (*Zea mays* cv. Kandy King) for these experiments since it had been used before to study the physiological effects of GLV and IE on maize defense responses [41,43-45]. In addition to the 6 genes listed above we also monitored transcript accumulation of CBP CCD1 (MZ00016998), calmodulin (MZ00019632), and allene oxide cyclase (MZ00024620) (Table 1) and we also expanded the time course from the microarray experiment to obtain more data on the temporal expression pattern of the selected genes. Based on the temporal pattern of transcript accumulation 3 distinct groups can be distinguished. WRKY12, a transcription factor was distinctly upregulated at 20 min, but declined rapidly thereafter. Likewise, CBP CCD1 was also only upregulated at 20 min after exposure (Figure 3). In contrast, calmodulin, MYC7, and lipase 2 showed maximum transcript accumulation at 60 min, whereas AOS, AOC, RIP, and also EREBP appear to level out between 60 and 120 min of exposure (Figure 3). 

**Figure 4:**
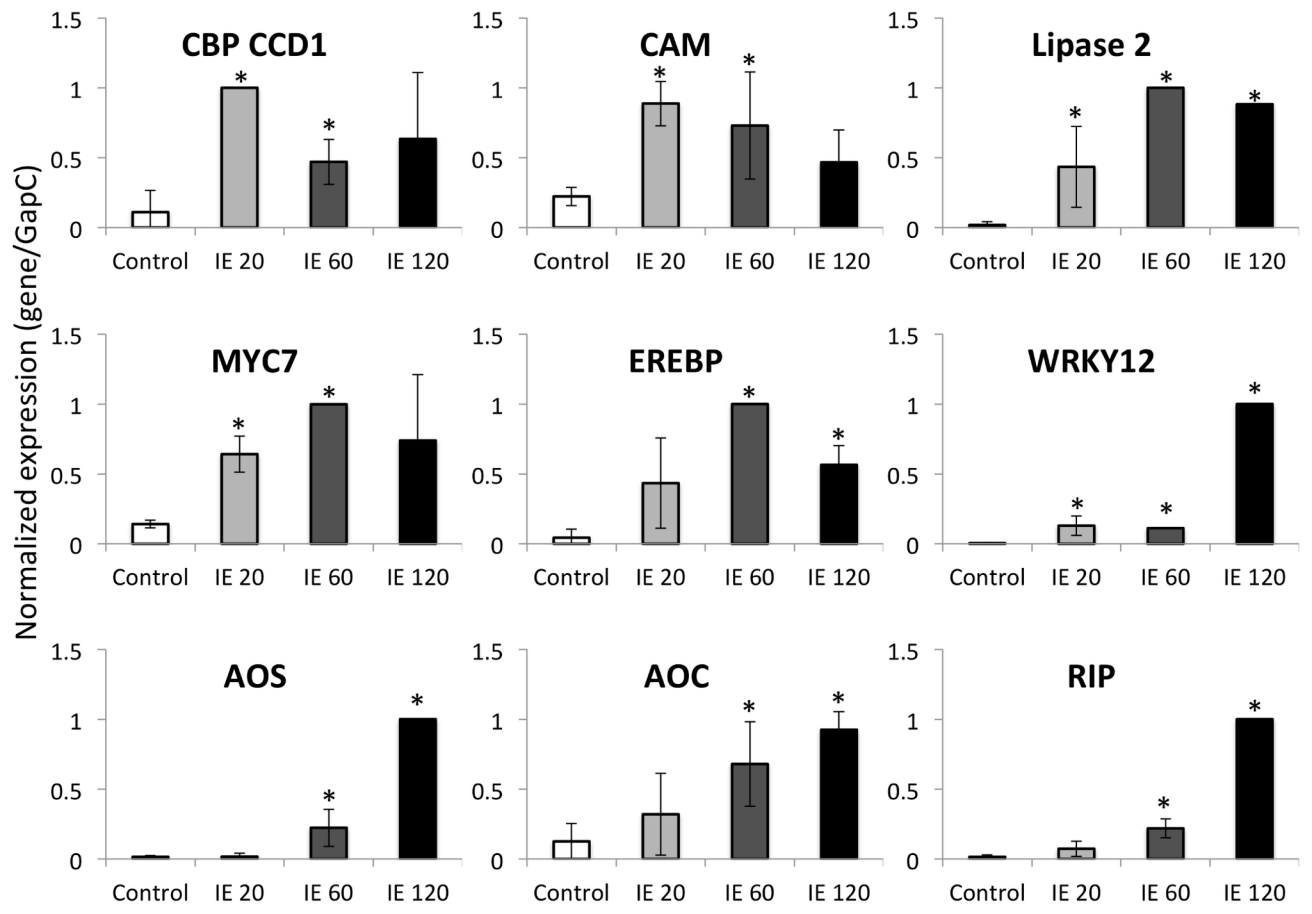
Transcript accumulation of selected genes after treatment with insect elicitor. Gene expression is displayed as relative abundance of transcripts in maize leaves (cv “Kandy King”) 20, 60, and 120 min after treatment. Data is expressed as the gene/GapC ratio. All data were normalized. Experiments have been performed with at least three biological replicates. A Student’s *t*-test was used for proof of significance (*, p≤0.05) when compared to the respective control. The lines on each bar represent standard deviation. Abbreviations: C, control; ie, insect elicitor (volicitin); AOS, allene oxide synthase; AOC, allene oxide cyclase; CBP CCD1, EF-hand Ca^2+^-binding protein CCD1; CAM, calmodulin; EREBP, ethylene response element binding protein; RIP, ribosome inactivating protein.

Since GLV have been described to have their major biological activity in the plant defense response against insect herbivores we also tested the 9 genes described above for their response to IE treatment (here: volicitin) over the same time course. While all 9 genes responded to volicitin treatment with enhanced transcript accumulation we also detected some differences in the temporal distribution of these transcripts. For example, AOS, AOC, WRKY12, and RIP appeared to increase most significantly between 60 and 120 min after treatment. Others like lipase 2 and CBP CCD1 maintained higher expression levels over a longer period of time (Figure 4). Mostly, however, with the exemption of WRKY12, gene expression induced by treatment with volicitin followed a similar general pattern when compared to *Z*-3-HOL treatment.

**Figure 5:**
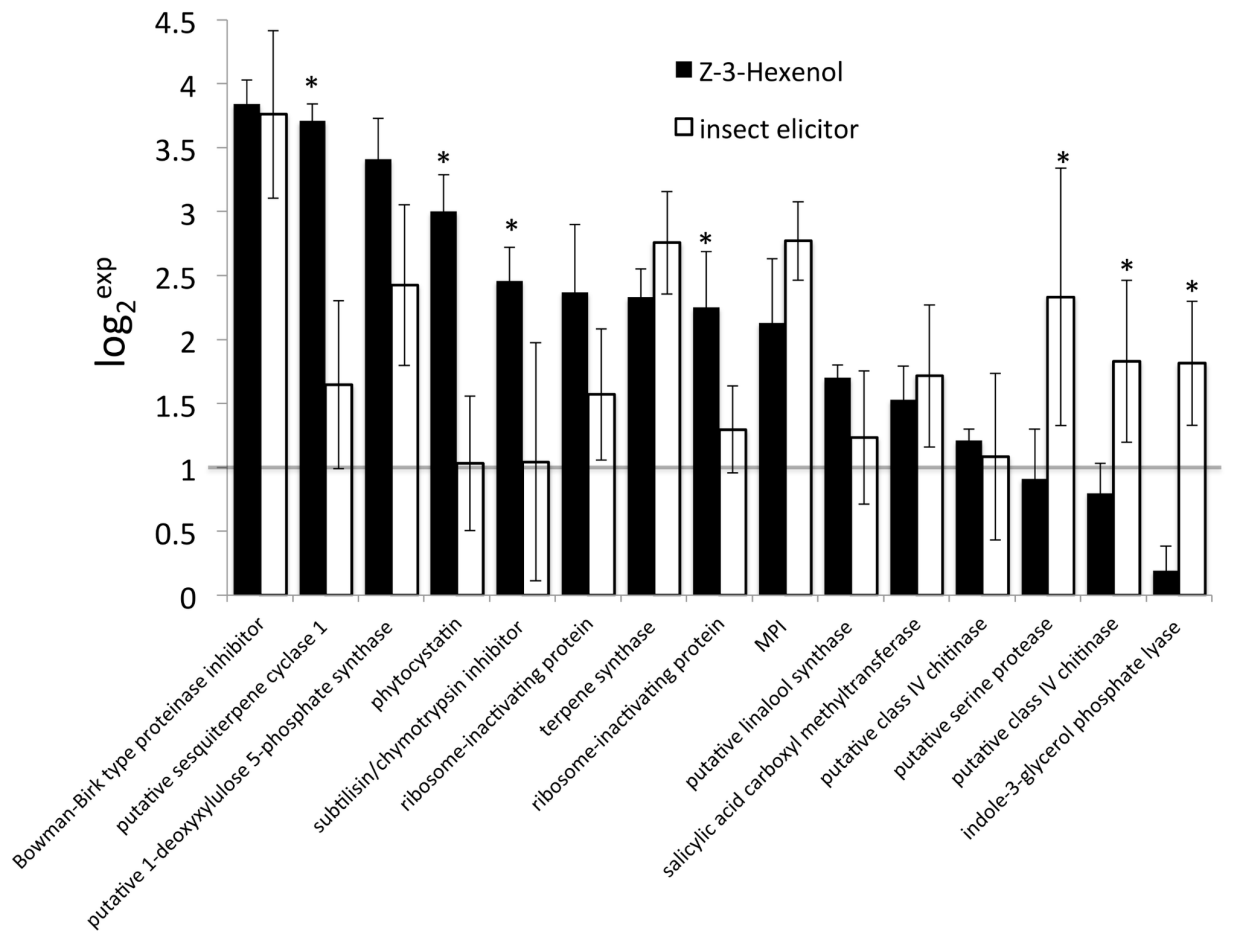
Microarray expression analysis of genes putatively involved in direct and indirect defenses at 60 min after treatment with Z-3-hexenol (black bars) and insect elicitor (volicitin) (white bars). The averages of log_2_-based increases of 3 independent microarrays (for Z-3-hexenol) and 4 independent microarrays for insect elicitor treatment are shown. At least one treatment for every pair displayed resulted in a significant increase of expression of ≥2-fold (p≤0.05). Error bars represent standard deviation. A Student’s *t*-test was used for proof of significance (*, p≤0.05) between Z-3-hexenol and insect elicitor treatment for individual genes.

To further compare *Z*-3-HOL induced gene expression with that after IE treatment we also performed a microarray analysis of volicitin-induced gene expression in B73 maize. However, this experiment was constrained due to the discontinuation of the platform by the Maize Microarray Project at the University of Arizona. Therefore, only 1 time point (60 min) was monitored and thus allows only for a limited assessment of IE-induced global changes in gene expression. We analyzed leaf segments including the elicitor application site that corresponded to those used for the *Z*-3-HOL-induced gene expression study. Two leaf segments were pooled for one biological replicate and a total of 4 biological replicates were performed. We identified 126 genes that were significantly altered in their expression (≥2-fold up- or down-regulated) at this time point (Table S3). Among those genes we identified 65 that were also upregulated after *Z*-3-HOL exposure. While the presence of almost 50% unknown genes makes it difficult to characterize major differences between *Z*-3-HOL and IE-induced gene expression, we noticed several differences that confirmed previous studies. For example, the gene for indole-3-glycerol lyase (Igl) (MZ00005958), which is a key enzyme in volatile indole biosynthesis, was 3.6-fold upregulated 1h after IE treatment, but not after exposure to *Z*-3-HOL. Likewise, the gene for ACC oxidase (MZ00039812), which produces ethylene, was 3.3-fold upregulated by ie, but not affected by *Z*-3-HOL. A high degree of correlation was however found by comparing genes involved in direct and indirect defenses including protease inhibitors and those responsible for HIPV production. When we compared transcript accumulation of these defense genes at the 1h time point based on the microarray data for both treatments we found that between the two treatments a total of 15 genes involved in the direct and indirect defense response against insect herbivores were induced (Figure 5). However, not all genes were induced by both treatments similarly. *Z*-3-HOL induced the significant accumulation of 12 defense genes at this time point, while volicitin-treatment only induced 9 of these genes. Only three defense genes including a putative serine protease (MZ00043994), a class IV chitinase (MZ00018180), and Igl (as mentioned above) were significantly higher induced in volicitin-treated maize seedling when compared to *Z*-3-HOL-exposed plants. This finding strongly suggests a high direct defense induction potential for *Z*-3-HOL. 

**Figure 6:**
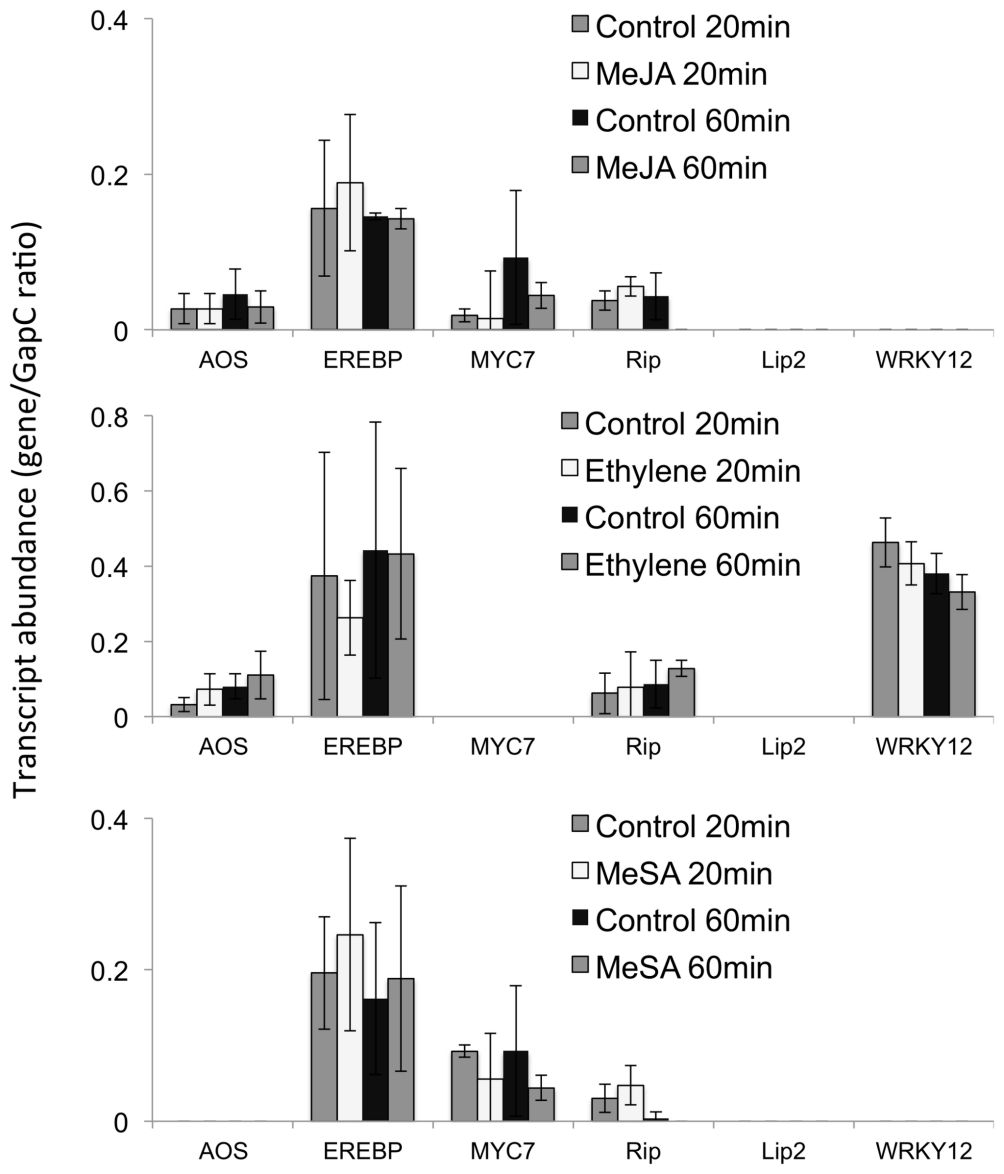
Comparison of defense gene expression in maize seedlings treated with methyl jasmonate, ethylene, and methyl salicylate. Gene expression is displayed as relative abundance of transcripts in maize leaves (Hybrid line “Kandy King”) 20 and 60 min after exposure to the respective volatile signaling compound. Data is expressed as the gene/GapC ratio. All experiments have been performed with at least three biological replicates. The lines on each bar represent standard deviation. Abbreviations: MeJA, methyl jasmonate; MeSA, methyl salicylate; AOS, allene oxide synthase; RIP, ribosome inactivating protein; EREBP, ethylene response element binding protein. No significant differences between treatment and control (Student’s *t*-test, P<0.05, *t*-test) were observed for any of the treatments.

While GLV are produced and released by all plant species tested to date after mechanical wounding and insect herbivory, other potential volatile signaling compounds are released in a more species-specific fashion and may include MeJA and MeSA among various other compounds. Since these two compounds in particular have been demonstrated to exhibit defense-inducing potential we also tested them for their activity on transcript accumulation of the 6 selected genes described above. In addition we tested E since it is usually also released by plants under insect herbivore attack. We used similar concentrations and followed the original time course (20 min and 60 min) of the microarray study as described above. Surprisingly, we could not detect any transcript accumulation for any of the selected genes over the selected time course (Figure 6) strongly suggesting that these volatile signals individually do not play a significant role in the regulation of anti-herbivore related plant defenses. 

## Discussion

In recent years green leaf volatiles (GLV) have emerged as important signals for plants in response to insect herbivory. GLV are released immediately after leaves get damaged by feeding insects and may serve as signals not only locally around the damage site, but also for other parts of the same plant as well as for other plants that are eavesdropping on their neighbor’s misery [4]. While physiological responses to GLV have mostly been described as incomplete or weak when compared to those induced by either actual herbivore damage or application of JA as the major regulator of defenses, they have nonetheless been demonstrated to have a significant impact on herbivore and pathogen performances as well as the physiological responses to insect-derived elicitors [22-27,34-37]. For the past 10 years the focus of research related to the biological activity of GLV was on their capacity to prime plants against insect herbivore damage resulting in a stronger and faster response to this kind of damage [23-27,33]. It was found that this priming effect was specifically herbivory-related, while for example responses to mechanical damage alone were not affected [33]. This priming effect has been shown for various plant species and was always specific with regard to the defensive action that was used by the respective plant. For example, maize produced more volatiles in response to IE treatment when plants were previously exposed to GLV [33] while Lima beans (*Phaseolus lunatus*) enhanced their extrafloral nectar production and *Nicotiana attenuata* produced more proteinase inhibitors [23,26,27]. From these and other studies it became obvious that GLV activity is tightly associated with the JA signaling pathway. However, even though GLV caused an immediate accumulation of JA in monocots, no such response was ever described for dicot plants despite the fact that they also showed a JA-dependent priming response [37,39]. While all this has been described repeatedly, there is still very little known about how GLV cause plants to activate their defenses. The goal of the project presented herein was therefore to further characterize the biological activity of *Z*-3-HOL as our model GLV by studying changes in global gene expression. Since GLV were found previously to induce JA accumulation very quickly [41], we focused on rapid changes in the transcriptome in an attempt to identify early regulatory elements activated by *Z*-3-HOL. By looking at transcriptome changes 20 and 60 min after exposure we could clearly identify 2 distinct tiers of transcriptional regulation indicated by the lack of overlapping genes between the two time points, in particular transcriptional regulators and defense genes. And although the absolute number of transcriptional regulators that are up at 60 min is higher, they represent only 10-15% of all significantly changed genes. In contrast, transcriptional regulators account for almost 50% at the early time point. While the functional role of these transcriptional regulators is still unknown, it must be assumed that these early regulators are serving as main switches for all subsequent metabolic remodeling through the activation of a second-tier level of genes. Interestingly, maize MYC7, a putative ortholog of the *Arabidopsis* MYC2, was found to belong to the second tier of transcriptional regulators, while in *Arabidopsis* it is definitely among the more rapidly induced genes [46]. There, MYC2 was found to be an essential regulator of JA-induced transcriptional changes, in particular those induced by mechanical wounding, and thus, is essential for defense regulation [46]. However, in a previous study on elicitor-induced distant signaling in maize we found that MYC7 transcripts did not accumulate after mechanical wounding alone, but required the presence of an IE to become activated [45]. In fact, IE treatment not only induced MYC7 accumulation at the damage site, but also in other undamaged parts of the plant [45]. 

Aside from transcriptional regulators the results of this microarray study clearly support the functional role of GLV in the wound and defense response of maize. Most, if not all relevant aspects of responses to these damaging events are to some degree covered by the genes activated by *Z*-3-HOL. For example, Ca^2+^ signaling has been reported previously to be an important factor in the regulation of plant defenses against insect herbivores and cytosolic Ca^2+^ levels were found to increase not only with insect herbivore damage, but also with the application of IE and also by exposing plants to GLV [47-51]. In accordance with these findings we identified several genes involved in Ca^2+^ signaling. For example, CBP CCD1 was upregulated already 20 min after exposure, while calmodulin, another important Ca^2+^-binding protein as well as a Ca^2+^-ATPase (MZ00047131) and two Ca^2+^-dependent protein kinase were significantly upregulated at 1 h. 

Mechanical damage as it occurs during insect herbivory already denotes a significant problem to the plant. For example, while without any mechanical barrier like the cuticle pathogens may succeed in invading the plant through the damage site thereby causing further damage. Additionally, a decrease in structural integrity of the wounded tissue together with uncontrollable water loss through the damage site comprise further challenges for the plant in its attempt to maintain physiological functionality. To counteract these effects plants have developed an array of mechanisms often resulting in the reinforcement of their cell walls and the fine-tuning of the cellular water potential. Important regulators in this process are proline- and glycine-rich proteins [52]. Both have been found to be inducible under various stresses including pathogen infection and drought stress. Proline-rich proteins are often secreted into the cell wall and can become cross-linked through reactive oxygen species by peroxidases thereby providing further stability to the cell wall [52-54]. Furthermore, they may serve as a scaffold for lignin depositions [55]. Both processes may be closely linked and as such may provide the damaged or neighboring cells with the means to maintain their structural integrity. As described above we have identified several proline- and glycine-rich proteins together with a key enzyme for lignin biosynthesis in maize seedlings exposed to *Z*-3-HOL. Since GLV are massively produced at the damage site it seems therefore natural that they may represent the major regulatory element in the process of reducing the impacts of mechanical damage. 

GLV have been shown in the past to have a significant impact on the plant defense against insect herbivores [4,5]. However, it has always been noted that this effect, mostly expressed as changes in defense-related gene expression, was significantly less pronounced or incomplete when compared to actual herbivory. However, by examining the data presented herein it appears as though many genes are actually induced very transiently by *Z*-3-HOL including, for example, CBP CCD1, lipase 2, calmodulin, WRKY12, and MYC7. Since in many of the previous studies about the biological activity of GLV and other volatiles much later time points ranging from 4 h to several days were studied [22,32], these and probably many other genes were likely missed. On the other hand we have shown herein also that some genes like Igl and ACC oxidase are only induced by ie, but not by GLV, supporting the notion that GLV in general or individual components thereof do indeed only induce subsets of anti-herbivore defenses. Nonetheless, this transient character of defense-related gene expression may have led ultimately to the discovery of a function for GLV aside from regulating direct defenses. Exposure to GLV appears to prime plants against subsequent insect herbivory by making them respond faster and stronger compared to un-primed controls. Priming is thought to have little impact on the general physiology since it does not necessarily require significant investments into defense. However, as described above, we found a massive induction of genes directly or indirectly involved in defenses. For example, the highest induction for any gene identified in this study was found for a Bowman-Birk proteinase inhibitor (14-fold upregulated) and at least 11 other related genes were found to be upregulated within 1h of exposure to *Z*-3-HOL. This strongly suggests that *Z*-3-HOL exposure, besides priming plants, may also has a more direct effect on insect herbivore performance. Likewise, a putative sesquiterpene cyclase was 13-fold upregulated, accompanied by other genes putatively involved in terpene volatile biosynthesis. While in the past these terpene-derived volatiles were shown to be released after GLV exposure [33,56,57], this was however always much lower when compared to actual insect herbivory. Also, indole-3-glycerol phosphate lyase (Igl), the key enzyme for volatile indole biosynthesis, was only induced by IE treatment, but not after exposure to *Z*-3-HOL. Indole is a major component of maize HIPV and often serves as a marker for this kind of damage. However, we have never observed any release of indole in maize seedlings treated with GLV nor have we ever observed any significant effect of GLV-induced priming on indole emission. It may therefore represent one of the few herbivore-specific responses that are regulated independent of GLV and are thus not “primable”. Since plants receiving these volatile signals are often not under attack yet, this reduced level of defense might simply be a result of the limited resources plants are willing to invest in defensive measures at this time. Nonetheless, the significant activation of volatile related genes by *Z*-3-HOL without a concomitant release of volatiles clearly shows that this indirect defense is a major target for GLV-mediated priming in maize, which has been shown previously on a metabolic level [33].

Based on these results we concluded that *Z*-3-HOL may have a much more essential function in regulating plant defenses than hitherto thought. This became even more obvious when we compared *Z*-3-HOL-induced gene expression with that of other potential volatile signaling compounds like E, MeSA, and MeJA. Surprisingly, none of these volatile signals exhibited any activity towards the activation of typical anti-herbivore defenses nor did they have any effect on herbivore performance. MeSA is released by maize seedling in small quantities when under herbivore attack and contributes to the typical bouquet of volatiles [58]. 

E is known to have synergistic effects on IE- and GLV-induced defense responses, but was never found to have a defensive function in maize itself [57,59,60]. While this strongly suggests that E produced after insect herbivore damage is not a defense signal itself but rather a general stress indicator that aids in the respective response, it nonetheless contributes significantly to the overall response. We identified several ethylene response element binding proteins (EREBP) to be significantly upregulated after *Z*-3-HOL and IE treatment. However, only IE treatment induced the accumulation of ACC oxidase, which is responsible for the production of E. This supports previous findings on the role of E in the regulation of anti-herbivore defense responses in maize. While GLV treatment has never been reported to induce the production of E, IE (including volicitin) treatment as well as actual insect herbivory induced E significantly in maize seedlings [6,59,60]. Furthermore, it was found that co-treatment of maize seedling with E and GLV or E with IE had strong synergistic effects on the responses to those two treatments [53,59,60]. Therefore, it appears as though GLV treatment prepares maize seedlings for increased perception of the E signal, but only actual herbivory also induces this compound. This may imply that the activation of E-responsive regulators might be part of the priming effects observed for GLV [33] by making those plants more sensitive to E. 

MeJA has been described repeatedly to induce genes likely to be involved in the plant defense response. In particular, *Arabidopsis* and tomato (*Solanum esculentum*) were found to respond to this volatile signal by activating proteinase inhibitors and other defense related proteins as well as toxic or repelling secondary metabolites. Likewise, in maize MeJA was found to induce a variety of genes involved in defense responses including Mir1, a cysteine protease that disrupts the peritrophic matrix in caterpillars [61], and Igl [62]. Also, MeJA induced the expression of a calcium dependent protein kinase (ZmCPK11), which is involved in wound signaling in maize [62,63]. These examples show that MeJA can be recognized by maize and induce defense-related genes. However, we found that MeJA had no effect on the defense genes selected for this study. While we cannot explain the discrepancy between these results we also noticed over years of volatile analyses that maize does not release MeJA as a volatile even when severely damaged by insect herbivores or treated with ie, which may reflect the limited responsiveness to this compound. 

By comparing these results it appears as though GLV are the most effective volatile defense signals in the maize system. Not only do they prime plants against insect herbivore attack, they may also provide effective direct protection for those plants in close proximity of the attacked emitter plant. 

In this study we identified more than 150 genes that are significantly up- or down-regulated within 1 h after exposure to *Z*-3-HOL. Among the upregulated genes we found many that are putatively involved in transcriptional regulation, Ca^2+^-signaling, lipid signaling, maintenance of structural integrity, and direct and indirect defenses against insect herbivores. Despite differences in the temporal distribution, a high degree of correlation was found between *Z*-3-HOL and IE-induced genes further supporting the important role that GLV play in the regulation of plant defense responses against insect herbivores in maize. 

## Materials and Methods

### Chemicals

*Z*-3-hexenol (*Z*-3-HOL) and jasmonic acid-methyl ester (MeJA) were obtained from Bedoukian Research (Danbury, CT, USA). Ethylene (E) was obtained from Fisher Scientific (Fisher Scientific Company LLC, Houston, TX ), and salicylic acid-methyl ester (MeSA) was purchased from Sigma Chemicals (St. Louis, MO). All solvents used were analytical grade. 

### Plant material

Maize seeds (*Zea mays* cv. B73, inbread line provided by Dr. M. Kolomiets, Texas A&M University; *Zea mays* cv. ‘Kandy King” J.W. Jung Seed Company, Randolph, WI) were grown in soil (Redi Earth Plug and Seedling Mix, Sun Gro) under 12h light/12h dark conditions with 60% relative humidity. Maize seedlings were used for experiments at the 2-leaf stage. 

### Plant treatments, RNA isolation, and Microarray-based global gene expression analysis

To study the physiological response expressed as transcript accumulation of maize plants to green leaf volatiles on a global scale 2-week-old intact maize seedlings (cv. B73; V2 stage) were exposed to *Z*-3-HOL as our model GLV. To avoid effects of solvent we used 1 mg of pure compound and added it to a paper strip attached to the inside of a 7-l glass cylinders. This corresponds to a maximum volatile concentration of approximately 1.4 µM in the gas phase. Controls were similarly treated but without the addition of *Z*-3-HOL. After 20 and 60 min of exposure the maize seedlings were removed from the cylinders and the second leaf was taken and immediately shock-frozen in liquid N_2_. Three segments per treatment were pooled for one biological replicate. A total of three biological replicates were collected per time point. 

For the microarray study on IE-induced changes in global gene expression we also used maize seedling (cv. B73) at the V2 stage as described above. The treatment of maize seedlings with pure IE (here: volicitin) was performed as described in [6]. In brief, an area of about 2mm x 10mm on the second leaf of intact maize plants was scratched with a razor blade and 10 µl of volicitin (in 50mM KPi, pH 8, corresponding to 1nmol of active compound) immediately added to the wounded site. Plants were treated for 60 min. Two segments of about 2.5 cm including the elicitor application area were then cut from the second leaf and pooled for one biological replicate. A total of 4 biological replicates were collected for microarray analysis. 

The pooled leaf material from B73 plants, as described above, was crushed and mixed with a sterile wooden stick, and 50-100 mg were taken from each biological replicate for RNA extraction. Total RNA was extracted with the Ultra Clean Plant RNA Isolation Kit (MO BIO Laboratories, Carlsbad, CA) according to the manufacture’s instructions with the following modifications. Frozen plant samples were homogenized in 2 ml screw cap FastPrep tubes containing 0.5 g of Zirmil microbeads and 200 µl extraction buffer (PR1) for 20 sec at 6000 rpm in a Precellys tissue homogenizer (MO BIO Laboratories, Carlsbad, CA). After this initial homogenization step 800 µl of PR1 were added and the sample again homogenized for 10 sec at 6000 rpm. The extract was then further processed as described in the manufacturer’s instructions. The extracted RNA was quantified and 20 µg of total RNA from each sample were taken and shipped overnight on dry ice to the Maize Microarray Project at the University of Arizona, where all subsequent steps including RNA labeling, hybridization, scanning, data extraction, and the normalization of data according to Loess were performed according to standard protocols (http://ag.arizona.edu/microarray/Microarraymethod1.doc). For the *Z*-3-HOL-induced gene expression study 3 biological replicates were performed including 1 dye swap, and for the volicitin-induced gene expression study 4 biological replicates including 2 dye swaps were performed. The data has been made available at the NCBI Gene Expression Omnibus (GEO accession numbers GSE47982 and GSE50981). 

### Treatment of maize seedlings for confirmational gene expression studies

The first conformational study for high *Z*-3-HOL-induced gene expression was also performed with B73 plants exactly as described above. For induction of maize (cv. Kandy King) seedlings with lower concentrations of *Z*-3-HOL, 10 µg (dissolved in dichloromethane, 1 µg/µl) were pipetted onto a cotton ball in the glass cylinder. This corresponds to a maximum concentration of approximately 14 nM of *Z*-3-HOL in the gas phase of the chamber. Controls consisted of a plant in a glass cylinder with 10 µl of pure dichloromethane on a cotton ball. As above, plants were removed from the glass cylinder after 20, 60, and 120 min, respectively, for further analysis. Also, we used a more open system by lifting the glass containers on spacers (about 2.5 cm high) to create an opening at the bottom part to allow for gas exchange. The second leaf of each seedling was then harvested and immediately shock-frozen in liq. N_2_ for further processing. Also, three leaves were pooled for one biological replicate. 

The treatment of maize seedlings (cv. Kandy King) with pure IE (volicitin) for PCR-based gene expression analysis was performed as described above for the microarray analysis. Plant were treated for 20, 60 and 120 min. Sections of about 2.5 cm including the elicitor application site were taken for further analysis and immediately shock-frozen in liq. N_2_. Plant material for one biological replicate was pooled from 3 treated plants. 

### Treatment of maize seedling with MeJA, MeSA, and E

To study the effect of MeJA, MeSA, and E on the activation of defense-related gene expression 2-week-old intact maize seedlings were exposed to these compounds as described above for treatment with Z-3-HOL in a 7-l glass cylinders. MeJA- and MeSA-treated plants were exposed to 1µl of pure compound, while E was applied as a gas at a final concentration of 4 ppm. Controls were done likewise, however without the addition of volatile signaling molecule. After 20 and 60 min of exposure the maize seedling were removed from the cylinders and the second leaf was taken and immediately shock-frozen in liquid N_2_. Three segments per treatment were pooled for one biological replicate. A total of three biological replicates were collected per time point.

### Confirmational gene expression analysis by semi-quantitative PCR

For confirmational studies as well as comparisons of Z-3-HOL-induced transcript accumulation with those observed after treatment with IE total RNA was extracted from app. 50-100 mg of the pooled maize leaves as described above. DNase treatment was performed with 3.125 µg total RNA with the Turbo DNA free kit (Ambion). For Reverse transcription 1.525 µg (in 12 µL water) of DNA-free RNA were mixed with 1 µl oligo dT´s (100 mM), 1µl oligo dTs (100 mM), 2 µl RT buffer (10x), 2 µl dNTP´s (5 mM each), 1 µl RNase inhibitor (10 U/µl), and 1 µl reverse transcriptase (5 U/µl) (Omniscript kit, Qiagen). The reaction mixture was incubated for 90 min at 37°C. For semi-quantitative analysis of gene expression, the cDNA was diluted (1:10) and 5 µl from this dilution was used for PCR. Primers were used as shown in Table 1. 

The PCR volume was 20 µl, containing 5X green GoTaq buffer (Promega) (4 µl), MgCl2 (25 mM, 1.2 µl), dNTP´s (25 mM, 0.32 µl), gene-specific primers (forward and reverse, 10 µM each, 2 µl), GapC primer (forward and reverse, 5 µM each, 2 µl), and GoTaq polymerase (5 U/µl, 0.2 µl). The volume was adjusted with DEPC water. Quantitative PCR was performed on a Eppendorf Mastercycler. The linear amplification range for each gene was first established by analyzing increases in gene band intensity in a 2.5% agarose gel after 22, 24, 26, 28, 30, and 32 cycles. Based on this information the following program was used for amplification: 95°C for 3 min, then (94°C for 30 sec, 54°C for 30 sec, 68°C for 1 min) x 27, then 68°C for 7 min for final extension. 10 µl of the PCR products were separated on a 2.5 % agarose gel for analysis. Ethidium-bromide stained bands were analyzed with a Photodyne documentation system and expression of genes was normalized by comparison with GapC. 

### Statistical analysis

At least three biological replicates of all experiments were performed. Microarray data were tested for significant expression using JMP 8.0 software (SAS Statistical Analysis Software, www.sas.com). At a significance level of p≤0.05, all genes were tested for 2-fold changes. A *t*-test was used to compare hybridization values. Based on a log_2_-scale a 2-fold increase or decrease would result in a hypothesized mean of ±1. Genes greater or equal to the hypothesized value were determined to be significantly up-regulated, and genes lesser or equal to the hypothesized value were determined to be significantly down-regulated. 

For the PCR-based expression analyses data was first normalized. A *t*-test was performed to confirm significant difference (p ≤ 0.05) between individual treatment groups and their respective controls. 

### Establishing annotations

Probe target sequences provided by the Maize Array Project were compared against the NCBI database using BLAST tools. Homologies higher than 80% according to NCBI databases were considered as a good homology.

## Supporting Information

Table S1 **Expression data of Z-3-hexenol-induced ESTs 20 min after exposure.**
(DOCX)

Table S2 **Expression data of Z-3-hexenol-induced ESTs 60 min after exposure.**
(DOCX)

Table S3 **Expression data of insect elicitor-induced ESTs 60 min after treatment.**
(DOCX)
