# Supplementary material for: Early Transcriptome Analyses of Z-3-Hexenol-Treated Zea mays Revealed Distinct Transcriptional Networks and Anti-Herbivore Defense Potential of Green Leaf Volatiles
Source: PLoS One. 2013 Oct 14;8(10):e77465. doi: 10.1371/journal.pone.0077465 (PMC3796489; doi:10.1371/journal.pone.0077465)
Supplement: Table S1 — Expression data of Z-3-hexenol-induced ESTs 20 min after exposure. (DOCX) [file pone.0077465.s001.docx]

Table S1

| ID | Name | Putative Annotation | Average | STDV | *p*-value 2-fold |
| --- | --- | --- | --- | --- | --- |
| MZ00017552 | TC273413 | Putative EREBP AP1-like | 2.15 | 0.26 | 0.0085 |
| MZ00029551 | TC252147 | Zinc-finger protein 1 | 2.09 | 0.21 | 0.0063 |
| MZ00022466 | TC279023 | ^†^ CAF1 family-like ribonuclease OSJNBa0088H09.11 | 1.94 | 0.19 | 0.0068 |
| MZ00005265 | BM381583 | ^†^ putative helix-loop-helix DNA-binding protein | 1.92 | 0.08 | 0.0013 |
| MZ00029177 | TC263931 | putative serine/threonine phosphatase type 2c | 1.92 | 0.29 | 0.0157 |
| MZ00042137 | TC195883 | phosphate-induced protein 1-like protein | 1.92 | 0.28 | 0.0155 |
| MZ00037057 | BM350776 | NA | 1.90 | 0.17 | 0.0063 |
| MZ00042093 | TC258542 | metallothionein 2a | 1.87 | 0.21 | 0.0097 |
| MZ00016998 | TC261915 | ^†^ EF-hand Ca2+-binding protein CCD1 | 1.83 | 0.31 | 0.0215 |
| MZ00017211 | TC252261 | ^†^ hypothetical protein | 1.81 | 0.19 | 0.0096 |
| MZ00032849 | TC278144 | putative phi-1 | 1.79 | 0.24 | 0.015 |
| MZ00005101 | BM351379 | Putative PRAS-rich protein | 1.74 | 0.11 | 0.0041 |
| MZ00020958 | TC255273 | Putative zink-finger protein | 1.69 | 0.25 | 0.0209 |
| MZ00044273 | TC252828 | NA | 1.59 | 0.37 | 0.0248 |
| MZ00022166 | TC268413 | ^†^ putative TPR-containing nuclear phosphoprotein | 1.50 | 0.20 | 0.0257 |
| MZ00023296 | TC266405 | unknown protein | 1.47 | 0.03 | 0.0007 |
| MZ00031018 | TC277131 | Putative ethylene responsive transcription factor 4 | 1.46 | 0.21 | 0.0109 |
| MZ00039367 | CD997985 | ^†^ NA | 1.38 | 0.02 | 0.0009 |
| MZ00022797 | TC268368 | NA | 1.32 | 0.02 | 0.0006 |
|  |  |  |  |  |  |
| MZ00032666 | TC278543 | OSJNBa0088A01.13 | -1.14 | 0.03 | 0.0173 |

(^†^, up-regulated by insect elicitor treatment at 60 min)
