# Supplementary material for: Early Transcriptome Analyses of Z-3-Hexenol-Treated Zea mays Revealed Distinct Transcriptional Networks and Anti-Herbivore Defense Potential of Green Leaf Volatiles
Source: PLoS One. 2013 Oct 14;8(10):e77465. doi: 10.1371/journal.pone.0077465 (PMC3796489; doi:10.1371/journal.pone.0077465)
Supplement: Table S2 — Expression data of Z-3-hexenol-induced ESTs 60 min after exposure. (DOCX) [file pone.0077465.s002.docx]

Table S2

| ID | Name | Putative Annotation | Average | STDV | *p*-value (2-fold) |
| --- | --- | --- | --- | --- | --- |
| MZ00037085 | BM379431 | ^§^ Bowman-Birk type proteinase inhibitor | 3.84 | 0.19 | 0.0007 |
| MZ00025768 | TC260885 | glutathione S-transferase GST 20 | 3.76 | 0.22 | 0.0011 |
| MZ00031736 | TC257320 | putative sesquiterpene cyclase 1 | 3.71 | 0.13 | 0.0004 |
| MZ00005052 | BM348519 | ^§^ Hypothetical protein | 3.47 | 0.21 | 0.0013 |
| MZ00055448 | TC200462 | ^§^ unknown protein | 3.42 | 0.35 | 0.0036 |
| MZ00032136 | TC277790 | ^§^ putative 1-deoxyxylulose 5-phosphate synthase | 3.41 | 0.32 | 0.003 |
| MZ00042935 | TC247937 | NA | 3.19 | 0.27 | 0.0025 |
| MZ00031271 | TC276645 | ^§^ Transcription Factor | 3.05 | 0.42 | 0.0071 |
| MZ00026471 | TC262217 | ^§^ anthranilate synthase alpha 1 subunit | 3.01 | 0.11 | 0.0005 |
| MZ00029560 | TC265167 | phytocystatin | 3.00 | 0.29 | 0.0037 |
| MZ00056802 | AZM4_32522 | leucoanthocyanidin dioxygenase-like protein | 2.98 | 0.29 | 0.0037 |
| MZ00021800 | TC255294 | putative transposable element Tip100 protein | 2.95 | 0.28 | 0.0036 |
| MZ00039764 | CF023702 | ^§^ NA | 2.82 | 0.31 | 0.0048 |
| MZ00044190 | TC271827 | ^§^ allene oxide synthase | 2.75 | 0.50 | 0.0008 |
| MZ00015701 | TC271549 | ^§^ putative lipoxygenase | 2.72 | 0.23 | 0.0031 |
| MZ00021783 | TC267188 | ^§^ NA | 2.67 | 0.19 | 0.0021 |
| MZ00036791 | TC254843 | ^§^ OSJNBa0079A21.19 | 2.61 | 0.29 | 0.0056 |
| MZ00018052 | TC251299 | putative AMP binding protein 1 | 2.52 | 0.28 | 0.0055 |
| MZ00041005 | TC191170 | subtilisin/chymotrypsin inhibitor | 2.46 | 0.26 | 0.0052 |
| MZ00012674 | CF630339 | ^§^ hypothetical protein | 2.44 | 0.37 | 0.0108 |
| MZ00015176 | TC271321 | ^§^ unknown protein | 2.44 | 0.13 | 0.0014 |
| MZ00036803 | BI396304 | hypothetical protein | 2.43 | 0.34 | 0.0093 |
| MZ00025289 | TC269977 | ^§^ putative deoxycytidine deaminase | 2.41 | 0.90 | 0.0571 |
| MZ00000792 | AI861154 | NA | 2.4 | 0.40 | 0.0135 |
| MZ00042324 | AZM4_112108 | ribosome-inactivating protein | 2.37 | 0.53 | 0.0238 |
| MZ00018568 | TC252910 | ^§^ NA | 2.37 | 0.22 | 0.0042 |
| MZ00042000 | TC194951 | ^§^ At5g04080 | 2.34 | 0.31 | 0.0089 |
| MZ00037253 | BM500738 | subtilisin/chymotrypsin inhibitor | 2.34 | 0.44 | 0.0172 |
| MZ00030501 | TC254689 | ^§^ terpene synthase {Zea mays;} | 2.33 | 0.22 | 0.0048 |
| MZ00044375 | TC264455 | hypothetical protein | 2.33 | 0.26 | 0.0065 |
| MZ00017300 | TC196604 | ^§^ unknown protein | 2.32 | 0.18 | 0.0032 |
| MZ00026739 | TC272635 | ^§^ putative lipase | 2.30 | 0.60 | 0.0319 |
| MZ00043393 | TC249180 | ^§^ 2-oxoglutarate-dependent oxygenase | 2.3 | 0.17 | 0.0031 |
| MZ00026661 | TC247999 | putative NAC-domain protein | 2.26 | 0.30 | 0.0091 |
| MZ00042678 | TC202729 | ribosome-inactivating protein | 2.25 | 0.44 | 0.0195 |
| MZ00003724 | BG265210 | OSJNBb0070J16.3 | 2.24 | 0.20 | 0.0045 |
| MZ00020230 | TC254236 | ^§^ epoxide hydrolase-like protein | 2.20 | 0.09 | 0.001 |
| MZ00038794 | CD963114 | NA | 2.16 | 0.43 | 0.0213 |
| MZ00043232 | TC267158 | putative glycine-rich protein | 2.14 | 0.08 | 0.0009 |
| MZ00017335 | TC262739 | ^§^ glutamine-fructose-6-phosphate transaminase 2 | 2.13 | 0.12 | 0.0021 |
| MZ00015910 | TC251004 | ^§^ S-like RNase | 2.13 | 0.19 | 0.0049 |
| MZ00033310 | TC255381 | ^§^ Maize proteinase inhibitor MPI | 2.13 | 0.50 | 0.0298 |
| MZ00018837 | TC263625 | MAPK6 | 2.12 | 0.04 | 0.0002 |
| MZ00039805 | CF029218 | ^§^ NA | 2.10 | 0.28 | 0.0111 |
| MZ00042245 | TC196906 | putative gamma-lyase | 2.09 | 0.46 | 0.028 |
| MZ00014350 | TC258325 | ^§^ putative JAZ protein | 2.09 | 0.05 | 0.0005 |
| MZ00041671 | TC269732 | ^§^ putative 60S ribosomal protein | 2.08 | 0.13 | 0.0026 |
| MZ00036743 | TC270193 | ^§^ adhesive/proline-rich protein | 2.06 | 0.15 | 0.0036 |
| MZ00046592 | AZM4_105060 | putative glutathione S-transferase | 2.05 | 0.35 | 0.0178 |
| MZ00024351 | TC249514 | B1358B12.20 | 2.05 | 0.03 | 0.0001 |
| MZ00041634 | TC270190 | ^§^ adhesive/proline-rich protein | 2.04 | 0.09 | 0.0014 |
| MZ00019970 | TC254432 | ^§^ unknown protein | 2.04 | 0.19 | 0.0059 |
| MZ00039367 | CD997985 | ^§^ NA | 2.04 | 0.40 | 0.0237 |
| MZ00016981 | TC251484 | unknown protein | 2.03 | 0.24 | 0.0092 |
| MZ00041484 | TC270690 | probable embryo-abundant protein | 2.03 | 0.42 | 0.0259 |
| MZ00014772 | TC269731 | ^§^ unknown protein | 2.02 | 0.23 | 0.0084 |
| MZ00055936 | TC254270 | OSJNBa0038O10.9 | 2.02 | 0.24 | 0.0094 |
| MZ00025068 | TC260808 | ^§^ unknown protein | 2.01 | 0.17 | 0.0047 |
| MZ00018574 | TC274267 | ethylene responsive element binding factor3 | 2.01 | 0.27 | 0.0118 |
| MZ00023441 | TC268809 | ^§^ S-adenosylmethionine synthetase 2 | 2.01 | 0.26 | 0.0107 |
| MZ00042242 | TC270194 | ^§^ adhesive/proline-rich protein | 2.00 | 0.08 | 0.0012 |
| MZ00017211 | TC252261 | ^§^ hypothetical protein | 1.98 | 0.09 | 0.0014 |
| MZ00026277 | TC251129 | ^§^ unknown protein | 1.98 | 0.24 | 0.01 |
| MZ00026538 | TC272736 | ^§^ Unknown protein | 1.96 | 0.29 | 0.0151 |
| MZ00026418 | TC209166 | ^§^ putative neutral invertase | 1.95 | 0.08 | 0.0012 |
| MZ00001468 | AW308703 | NA | 1.94 | 0.30 | 0.0162 |
| MZ00013773 | TC269981 | putative UDP-glucose dehydrogenase | 1.93 | 0.24 | 0.0113 |
| MZ00035740 | AW360627 | ^§^ NA | 1.92 | 0.54 | 0.0492 |
| MZ00037127 | BM380742 | ^§^ NA | 1.92 | 0.21 | 0.009 |
| MZ00025872 | TC272087 | ^§^ NA | 1.91 | 0.53 | 0.0486 |
| MZ00035750 | AW400335 | ^§^ NA | 1.89 | 0.21 | 0.0097 |
| MZ00016430 | TC262362 | ^§^ NA | 1.88 | 0.42 | 0.0346 |
| MZ00036774 | TC272661 | putative gamma-lyase | 1.88 | 0.31 | 0.0201 |
| MZ00042477 | TC263172 | P-type ATPase | 1.87 | 0.04 | 0.0004 |
| MZ00036758 | BI097654 | hypothetical protein | 1.86 | 0.33 | 0.0239 |
| MZ00015177 | TC271323 | unknown protein | 1.85 | 0.10 | 0.0023 |
| MZ00018836 | TC263738 | ^§^ OSJNBa0079A21.19 | 1.85 | 0.12 | 0.0036 |
| MZ00001353 | AW231877 | NA | 1.82 | 0.19 | 0.0092 |
| MZ00030502 | TC254958 | ^§^ dioxygenase (Y09113) | 1.82 | 0.40 | 0.0356 |
| MZ00026392 | TC271619 | ^§^ Bax inhibitor-1 | 1.81 | 0.08 | 0.002 |
| MZ00014875 | TC260554 | putative RNA-binding protein | 1.81 | 0.06 | 0.0012 |
| MZ00054995 | PUDGD70TD | NA | 1.81 | 0.16 | 0.0069 |
| MZ00037883 | TC248976 | ^§^ 17.7 kDa low temperature induced protein | 1.79 | 0.15 | 0.0063 |
| MZ00023975 | TC275356 | unknown protein | 1.78 | 0.54 | 0.0452 |
| MZ00047131 | AZM4_11757 | type IIB calcium ATPase | 1.78 | 0.12 | 0.0042 |
| MZ00043117 | TC250981 | ^§^ OSJNBa0008M17.8 | 1.78 | 0.10 | 0.0031 |
| MZ00018741 | TC274949 | ^§^ putative cytochrome P450 reductase | 1.73 | 0.04 | 0.0007 |
| MZ00040654 | NP288639\| | glutathione S-transferase GST 25 | 1.72 | 0.38 | 0.0411 |
| MZ00056783 | AZM4_26485 | ^§^ unnamed protein product | 1.71 | 0.18 | 0.0106 |
| MZ00026596 | TC251139 | ^§^ ethylene responsive element binding factor3 | 1.71 | 0.25 | 0.0209 |
| MZ00019729 | TC254629 | putative linalool synthase | 1.70 | 0.10 | 0.0033 |
| MZ00021914 | TC256655 | ^§^ DNA-binding protein family-like | 1.70 | 0.06 | 0.0014 |
| MZ00041213 | TC191716 | putative UDP-glucose dehydrogenase | 1.7 | 0.05 | 0.0009 |
| MZ00028280 | TC263204 | calcium-dependent protein kinase | 1.65 | 0.19 | 0.015 |
| MZ00031957 | TC255210 | unknown protein | 1.64 | 0.24 | 0.0234 |
| MZ00023228 | TC278579 | ^§^ putative cinnamoyl-CoA reductase | 1.63 | 0.04 | 0.001 |
| MZ00025708 | TC271892 | unknown protein | 1.62 | 0.13 | 0.0074 |
| MZ00022466 | TC279023 | ^§^ CAF1 family-like ribonuclease | 1.60 | 0.08 | 0.0032 |
| MZ00000946 | TC257928 | hypothetical protein | 1.6 | 0.23 | 0.0229 |
| MZ00015033 | PUHKV48TB | NA | 1.57 | 0.09 | 0.0046 |
| MZ00023780 | TC269933 | ^§^ unnamed protein product | 1.57 | 0.30 | 0.0132 |
| MZ00018167 | TC273799 | OSJNBa0013K16.15 | 1.57 | 0.21 | 0.0209 |
| MZ00014943 | TC260783 | anthranilate synthase alpha 2 | 1.56 | 0.27 | 0.0343 |
| MZ00021703 | TC256836 | NA | 1.55 | 0.23 | 0.0275 |
| MZ00004877 | TC278806 | putative cytochrome P450 | 1.54 | 0.22 | 0.027 |
| MZ00023938 | TC270341 | phosphoenolpyruvate carboxylase | 1.54 | 0.13 | 0.0096 |
| MZ00032043 | TC278055 | ^§^ putative benzothiadiazole-induced S-adenosyl-L-methionine:salicylic acid carboxyl methyltransferase 1 | 1.53 | 0.26 | 0.0364 |
| MZ00015962 | TC261629 | putative choline kinase | 1.52 | 0.27 | 0.0402 |
| MZ00024784 | TC260606 | putative acyl-ACP thioesterase | 1.50 | 0.26 | 0.041 |
| MZ00019886 | TC248865 | ^§^ transcription factor MYC7E | 1.49 | 0.11 | 0.0082 |
| MZ00029456 | TC264850 | putative hexokinase 1 | 1.49 | 0.15 | 0.0151 |
| MZ00043996 | TC271620 | ^§^ Bax inhibitor-1 | 1.47 | 0.09 | 0.0066 |
| MZ00042567 | TC276563 | putative AAA-type ATPase | 1.47 | 0.23 | 0.0365 |
| MZ00019894 | TC274718 | ^§^ putative acid phosphatase | 1.46 | 0.21 | 0.0313 |
| MZ00029217 | TC264013 | unknown protein | 1.45 | 0.22 | 0.0374 |
| MZ00032405 | TC278495 | OSJNBa0014K14.7 | 1.44 | 0.25 | 0.0482 |
| MZ00044135 | TC251573 | putative JAZ protein | 1.44 | 0.03 | 0.0008 |
| MZ00044516 | TC253900 | NA | 1.43 | 0.11 | 0.0105 |
| MZ00015240 | TC261057 | putative soluble inorganic pyrophosphatase | 1.43 | 0.12 | 0.0128 |
| MZ00023547 | TC248274 | putative aminotransferase class-III | 1.42 | 0.10 | 0.0096 |
| MZ00017601 | TC272080 | hydroperoxide lyase {Zea mays;} | 1.41 | 0.12 | 0.014 |
| MZ00003755 | BG316497 | NA | 1.41 | 0.18 | 0.029 |
| MZ00042217 | TC273279 | unknown protein | 1.40 | 0.23 | 0.0498 |
| MZ00042105 | TC261136 | putative glucosyltransferase | 1.36 | 0.19 | 0.0396 |
| MZ00017669 | TC248988 | ^§^ Clp protease ATP-binding subunit | 1.36 | 0.10 | 0.0138 |
| MZ00027499 | TC252071 | putative quercetin 3-O-glucoside-6''-O-malonyltransferase | 1.35 | 0.15 | 0.0295 |
| MZ00019172 | TC263777 | putative katanin | 1.31 | 0.13 | 0.0278 |
| MZ00033116 | TC268314 | unknown protein | 1.28 | 0.09 | 0.017 |
| MZ00003659 | BE639046 | ^§^ lysine decarboxylase-like protein | 1.28 | 0.14 | 0.0376 |
| MZ00005265 | BM381583 | ^§^ putative helix-loop-helix DNA-binding protein | 1.27 | 0.14 | 0.0396 |
| MZ00042953 | TC248400 | chlorophyll a/b binding protein | 1.26 | 0.03 | 0.0032 |
| MZ00025951 | TC272271 | NA | 1.26 | 0.14 | 0.0432 |
| MZ00021345 | TC250281 | unknown protein | 1.24 | 0.07 | 0.0133 |
| MZ00021965 | TC267861 | hypothetical protein F2K15.100 | 1.22 | 0.11 | 0.0391 |
| MZ00052288 | BE509620 | ^§^ NA | 1.22 | 0.11 | 0.0406 |
| MZ00036158 | BE123246 | putative class IV chitinase | 1.21 | 0.09 | 0.0281 |
| MZ00028773 | TC274616 | receptor protein kinase PERK1-like protein | 1.19 | 0.03 | 0.0052 |
| MZ00044579 | TC254550 | putative protein kinase | 1.19 | 0.11 | 0.0483 |
| MZ00028873 | TC274036 | ^§^ putative mitochondrial carrier protein | 1.17 | 0.08 | 0.0374 |
| MZ00050523 | AZM4_60075 | putative calcium-dependent protein kinase | 1.16 | 0.07 | 0.0288 |
| MZ00000537 | AI649800 | putative TCP transcription factor | 1.15 | 0.06 | 0.0287 |
| MZ00039478 | CF004440 | oxysterol-binding protein | 1.07 | 0.02 | 0.0131 |
|  |  |  |  |  |  |
| MZ00018952 | TC253695 | hypothetical protein | -1.26 | 0.05 | 0.0067 |
| MZ00018923 | TC198574 | putative arabinogalactan protein | -1.37 | 0.23 | 0.05 |
| MZ00051085 | AZM4_70615 | tyrosine-specific protein phosphatase protein | -1.37 | 0.20 | 0.0417 |
| MZ00056066 | TC255466 | NA | -1.40 | 0.08 | 0.0069 |
| MZ00044026 | TC259103 | NA | -1.61 | 0.13 | 0.0079 |
| MZ00044027 | TC259106 | NA | -1.67 | 0.19 | 0.0132 |
| MZ00024420 | TC248752 | CIPK-like protein | -1.68 | 0.14 | 0.0073 |
| MZ00018192 | TC248950 | ^§^ beta-expansin 6 | -1.70 | 0.34 | 0.0358 |
| MZ00015576 | TC250773 | tyrosine-specific protein phosphatase protein | -1.72 | 0.31 | 0.0293 |
| MZ00015575 | TC250774 | tyrosine-specific protein phosphatase protein | -1.93 | 0.19 | 0.0071 |

(^§^, up-regulated by insect elicitor treatment at 60 min)
