# Supplementary material for: Early Transcriptome Analyses of Z-3-Hexenol-Treated Zea mays Revealed Distinct Transcriptional Networks and Anti-Herbivore Defense Potential of Green Leaf Volatiles
Source: PLoS One. 2013 Oct 14;8(10):e77465. doi: 10.1371/journal.pone.0077465 (PMC3796489; doi:10.1371/journal.pone.0077465)
Supplement: Table S3 — Expression data of insect elicitor-induced ESTs 60 min after treatment. (DOCX) [file pone.0077465.s003.docx]

Table S3

| ID | Name | Putative_Annotation | Average | STDV | *p*-value (2-fold) |
| --- | --- | --- | --- | --- | --- |
| MZ00055109 | AZM4_68925 | unknown protein | 4.32 | 0.45 | 0.000 |
| MZ00021783 | TC267188 | ^§^ NA | 3.66 | 0.49 | 0.001 |
| MZ00037085 | BM379431 | ^§^ Bowman-Birk type proteinase inhibitor | 3.59 | 0.66 | 0.002 |
| MZ00018741 | TC274949 | ^§^ putative cytochrome P450 reductase | 3.44 | 0.67 | 0.004 |
| MZ00055448 | TC200462 | ^§^ unknown protein | 3.39 | 0.58 | 0.002 |
| MZ00005052 | BM348519 | ^§^ Hypothetical protein | 3.37 | 0.18 | 0.000 |
| MZ00026471 | TC262217 | ^§^ anthranilate synthase alpha 1 subunit | 3.36 | 0.47 | 0.001 |
| MZ00015701 | TC271549 | ^§^ putative lipoxygenase | 3.24 | 0.68 | 0.006 |
| MZ00044190 | TC271827 | ^§^ allene oxide synthase | 3.21 | 0.28 | 0.000 |
| MZ00031823 | TC267413 | NPK1-related protein kinase-like protein | 3.10 | 0.43 | 0.001 |
| MZ00026538 | TC272736 | ^§^ Unknown protein | 3.05 | 0.58 | 0.004 |
| MZ00016998 | TC261915 | ^†^ EF-hand Ca2+-binding protein CCD1 | 2.94 | 1.04 | 0.014 |
| MZ00033310 | TC255381 | ^§^ Maize proteinase inhibitor MPI | 2.91 | 0.31 | 0.001 |
| MZ00025068 | TC260808 | ^§^ unknown protein | 2.88 | 0.48 | 0.003 |
| MZ00036791 | TC254843 | ^§^ OSJNBa0079A21.19 | 2.87 | 0.37 | 0.001 |
| MZ00039805 | CF029218 | ^§^ NA | 2.86 | 0.24 | 0.000 |
| MZ00042000 | TC194951 | ^§^ At5g04080 | 2.86 | 0.99 | 0.016 |
| MZ00030501 | TC254689 | ^§^ terpene synthase | 2.83 | 0.40 | 0.002 |
| MZ00031271 | TC276645 | ^§^ transcription factor | 2.81 | 0.70 | 0.005 |
| MZ00039764 | CF023702 | ^§^ NA | 2.81 | 0.61 | 0.005 |
| MZ00026277 | TC251129 | ^§^ unknown protein | 2.74 | 0.32 | 0.001 |
| MZ00025289 | TC269977 | ^§^ putative deoxycytidine deaminase | 2.73 | 0.83 | 0.013 |
| MZ00041671 | TC269732 | ^§^ putative 60S ribosomal protein | 2.63 | 0.29 | 0.001 |
| MZ00017335 | TC262739 | ^§^ glutamine-fructose-6-phosphate transaminase 2 | 2.62 | 0.33 | 0.001 |
| MZ00032136 | TC277790 | ^§^ putative 1-deoxyxylulose 5-phosphate synthase | 2.60 | 0.63 | 0.010 |
| MZ00041634 | TC270190 | ^§^ adhesive/proline-rich protein homolog-like protein | 2.56 | 0.54 | 0.004 |
| MZ00005095 | BM351040 | NA | 2.55 | 0.73 | 0.018 |
| MZ00012674 | CF630339 | ^§^ hypothetical protein | 2.53 | 0.52 | 0.008 |
| MZ00017211 | TC252261 | ^† §^ hypothetical protein | 2.50 | 0.31 | 0.001 |
| MZ00022466 | TC279023 | ^† §^ CAF1 family-like ribonuclease | 2.50 | 0.46 | 0.005 |
| MZ00036743 | TC270193 | ^§^ adhesive/proline-rich protein homolog-like protein | 2.47 | 0.45 | 0.003 |
| MZ00026418 | TC209166 | ^§^ putative neutral invertase | 2.44 | 0.48 | 0.006 |
| MZ00039367 | CD997985 | ^† §^ NA | 2.36 | 0.08 | 0.000 |
| MZ00027073 | TC272780 | OSJNBa0086B14.2 | 2.35 | 0.78 | 0.026 |
| MZ00005265 | BM381583 | ^† §^ putative helix-loop-helix DNA-binding protein | 2.33 | 0.21 | 0.001 |
| MZ00018568 | TC252910 | ^§^ NA | 2.30 | 0.37 | 0.004 |
| MZ00017300 | TC196604 | ^§^ unknown protein | 2.30 | 0.20 | 0.001 |
| MZ00005101 | BM351379 | NA | 2.30 | 0.87 | 0.022 |
| MZ00018137 | TC274363 | NA | 2.28 | 0.73 | 0.016 |
| MZ00017669 | TC248988 | ^§^ ATP-dependent Clp protease ATP-binding subunit precursor | 2.28 | 0.70 | 0.017 |
| MZ00038625 | CD955299 | NA | 2.27 | 1.16 | 0.049 |
| MZ00043994 | TC272489 | Putative serine protease | 2.25 | 1.01 | 0.038 |
| MZ00024350 | TC249515 | anthranilate synthase | 2.25 | 0.65 | 0.031 |
| MZ00057056 | AZM4_59590 | Unknown protein | 2.25 | 0.31 | 0.001 |
| MZ00019970 | TC254432 | ^§^ unknown protein | 2.23 | 0.59 | 0.011 |
| MZ00026739 | TC272635 | ^§^ putative lipase | 2.21 | 0.65 | 0.026 |
| MZ00021508 | TC201896 | chitin-inducible gibberellin-responsive protein | 2.21 | 1.12 | 0.047 |
| MZ00057335 | AZM4_93694 | putative calcium-dependent protein kinase | 2.20 | 0.97 | 0.037 |
| MZ00026596 | TC251139 | ^§^ ethylene responsive element binding factor3 | 2.16 | 0.60 | 0.019 |
| MZ00033132 | TC276030 | putative pirin | 2.15 | 0.31 | 0.002 |
| MZ00023343 | TC268633 | NA | 2.15 | 0.58 | 0.030 |
| MZ00043996 | TC271620 | ^§^ Bax inhibitor-1 | 2.15 | 0.32 | 0.002 |
| MZ00015910 | TC251004 | ^§^ S-like RNase | 2.14 | 0.83 | 0.020 |
| MZ00039806 | CF029488 | anthranilate synthase | 2.12 | 0.59 | 0.031 |
| MZ00043117 | TC250981 | ^§^ OSJNBa0008M17.8 | 2.10 | 0.79 | 0.027 |
| MZ00042242 | TC270194 | ^§^ adhesive/proline-rich protein homolog-like protein | 2.09 | 0.31 | 0.003 |
| MZ00005245 | BM381087 | NPK1-related protein kinase-like protein | 2.08 | 0.25 | 0.002 |
| MZ00043425 | TC249104 | NA | 2.08 | 0.69 | 0.025 |
| MZ00055049 | PUFXO20TBC | putative ATP-dependent proteinase | 2.07 | 0.39 | 0.009 |
| MZ00023501 | TC258147 | unknown protein | 2.07 | 0.33 | 0.006 |
| MZ00039734 | CF021235 | OSJNBb0070J16.3 | 2.05 | 0.36 | 0.006 |
| MZ00041736 | TC270416 | hypothetical protein 1 | 2.05 | 0.76 | 0.025 |
| MZ00041735 | BQ618980 | NA | 2.04 | 0.78 | 0.022 |
| MZ00014350 | TC258325 | ^§^ putative JAZ protein | 2.04 | 0.45 | 0.017 |
| MZ00014772 | TC269731 | ^§^ unknown protein | 2.03 | 0.34 | 0.003 |
| MZ00028898 | TC264560 | hypothetical protein | 2.02 | 0.39 | 0.012 |
| MZ00023789 | TC258613 | putative shaggy related protein kinase ASK-gamma | 2.01 | 0.47 | 0.018 |
| MZ00018836 | TC263738 | ^§^ OSJNBa0079A21.19 | 2.00 | 0.54 | 0.031 |
| MZ00032043 | TC278055 | ^§^ putative benzothiadiazole-induced S-adenosyl-L-methionine:salicylic acid carboxyl methyltransferase 1 | 1.97 | 0.56 | 0.041 |
| MZ00043120 | TC258657 | shaggy protein kinase 4 | 1.96 | 0.33 | 0.006 |
| MZ00033715 | TC276898 | hypothetical protein | 1.96 | 0.35 | 0.004 |
| MZ00025407 | TC260936 | Polyamine oxidase precursor | 1.94 | 0.25 | 0.003 |
| MZ00020525 | TC251574 | unknown protein | 1.93 | 0.18 | 0.001 |
| MZ00037137 | BM381169 | putative B12D protein | 1.92 | 0.49 | 0.016 |
| MZ00056783 | AZM4_26485 | ^§^ hypothetical protein | 1.92 | 0.50 | 0.028 |
| MZ00005958 | TC253202 | indole-3-glycerol phosphate lyase | 1.86 | 0.48 | 0.020 |
| MZ00018291 | TC273680 | NA | 1.86 | 0.88 | 0.050 |
| MZ00024602 | TC260212 | allene oxide cyclase | 1.85 | 0.39 | 0.017 |
| MZ00026392 | TC271619 | ^§^ Bax inhibitor-1 | 1.84 | 0.50 | 0.044 |
| MZ00019894 | TC274718 | ^§^ putative acid phosphatase | 1.83 | 0.50 | 0.049 |
| MZ00017546 | TC252802 | NA | 1.83 | 0.54 | 0.046 |
| MZ00023228 | TC278579 | ^§^ putative cinnamoyl-CoA reductase | 1.82 | 0.25 | 0.005 |
| MZ00035065 | AI396236 | NA | 1.81 | 0.69 | 0.042 |
| MZ00037883 | TC248976 | ^§^ 17.7 kDa low temperature induced protein | 1.80 | 0.28 | 0.005 |
| MZ00043393 | TC249180 | ^§^ 2-oxoglutarate-dependent oxygenase | 1.79 | 0.31 | 0.007 |
| MZ00030024 | TC273084 | putative WRKY transcription factor | 1.78 | 0.41 | 0.029 |
| MZ00052288 | BE509620 | ^§^ NA | 1.78 | 0.21 | 0.002 |
| MZ00017265 | TC252555 | MtN3-like protein | 1.78 | 0.46 | 0.049 |
| MZ00005985 | BQ619125 | AAA-type ATPase-like | 1.76 | 0.37 | 0.021 |
| MZ00035750 | AW400335 | ^§^ NA | 1.76 | 0.50 | 0.017 |
| MZ00023780 | TC269933 | ^§^ hypothetical protein | 1.74 | 0.31 | 0.011 |
| MZ00039812 | TC262946 | acc oxidase | 1.73 | 0.21 | 0.005 |
| MZ00036928 | BM080815 | unknown protein | 1.73 | 0.50 | 0.026 |
| MZ00016430 | TC262362 | ^§^ NA | 1.72 | 0.57 | 0.048 |
| MZ00004614 | TC271645 | NA | 1.70 | 0.48 | 0.029 |
| MZ00026064 | TC272222 | NA | 1.70 | 0.35 | 0.009 |
| MZ00021254 | TC266778 | OSJNBa0065B15.8 | 1.70 | 0.54 | 0.047 |
| MZ00028873 | TC274036 | ^§^ putative mitochondrial carrier protein | 1.70 | 0.51 | 0.036 |
| MZ00019886 | TC248865 | ^§^ transcription factor MYC7E | 1.69 | 0.20 | 0.005 |
| MZ00018009 | TC263399 | NA | 1.68 | 0.44 | 0.027 |
| MZ00037636 | CA402624 | 2-oxoglutarate-dependent oxygenase | 1.67 | 0.49 | 0.043 |
| MZ00025872 | TC272087 | ^§^ NA | 1.66 | 0.42 | 0.017 |
| MZ00023441 | TC268809 | ^§^ S-adenosylmethionine synthetase 2 | 1.65 | 0.33 | 0.016 |
| MZ00037127 | BM380742 | ^§^ NA | 1.63 | 0.41 | 0.047 |
| MZ00015176 | TC271321 | ^§^ unknown protein | 1.63 | 0.40 | 0.017 |
| MZ00021734 | TC256261 | NA | 1.62 | 0.06 | 0.000 |
| MZ00018180 | TC248737 | putative class IV chitinase | 1.60 | 0.63 | 0.040 |
| MZ00030502 | TC254958 | ^§^ dioxygenase | 1.57 | 0.60 | 0.048 |
| MZ00031310 | TC266681 | putative calmodulin | 1.55 | 0.58 | 0.047 |
| MZ00018342 | TC274315 | putative esterase | 1.52 | 0.49 | 0.042 |
| MZ00036171 | BE186250 | putative UDP-glucose dehydrogenase | 1.49 | 0.37 | 0.038 |
| MZ00029568 | TC253835 | Unknown protein | 1.49 | 0.27 | 0.022 |
| MZ00035740 | AW360627 | ^§^ NA | 1.48 | 0.38 | 0.022 |
| MZ00020230 | TC254236 | ^§^ epoxide hydrolase-like protein | 1.46 | 0.28 | 0.019 |
| MZ00028246 | TC248214 | GRAB1 protein | 1.43 | 0.35 | 0.029 |
| MZ00003659 | BE639046 | ^§^ lysine decarboxylase-like protein | 1.39 | 0.10 | 0.003 |
| MZ00035947 | AZM4_81598 | putative NAC-domain protein | 1.33 | 0.19 | 0.013 |
| MZ00019618 | TC264039 | unknown protein | 1.31 | 0.08 | 0.002 |
| MZ00033340 | TC278720 | NA | 1.31 | 0.10 | 0.004 |
| MZ00039846 | CF033245 | NA | 1.29 | 0.23 | 0.027 |
| MZ00025654 | TC271901 | unknown protein | 1.23 | 0.12 | 0.027 |
| MZ00030629 | TC265536 | NA | 1.14 | 0.13 | 0.028 |
|  |  |  |  |  |  |
| MZ00022590 | TC256960 | putative NAC domain protein | -1.268 | 0.560 | 0.05 |
| MZ00018192 | TC248950 | ^§^ beta-expansin 6 | -1.403 | 0.372 | 0.05 |
| MZ00044002 | TC251578 | licheninase | -1.635 | 0.395 | 0.016 |
| MZ00043843 | TC271864 | putative ZmGR1a | -2.408 | 0.944 | 0.035 |

(^†^, up-regulated by Z-3-hexenol exposure at 20 min, ^§^, up-regulated by Z-3-hexenol exposure at 60 min).
